# Second-line treatment strategies of ulcerative colitis after conventional therapy failure: A systematic review and network meta-analysis of randomized controlled trials

**DOI:** 10.1371/journal.pone.0337222

**Published:** 2025-12-01

**Authors:** Yanan Xu, Wenshuo Jiang, Meizhu Jiang, Bin Zhu, Jiping Huo, Mingfen Wu, Li Yang

**Affiliations:** 1 Department of Pharmacy, Beijing Tiantan Hospital, Capital Medical University, Beijing, P.R. China; 2 School of Pharmaceutical Sciences, Capital Medical University, Beijing, P.R. China; University of Diyala College of Medicine, IRAQ

## Abstract

**Purpose:**

This study aimed to compare the efficacy and safety of pharmacotherapies for ulcerative colitis (UC), promoting more precise management of refractory ulcerative colitis.

**Methods:**

Relevant randomized controlled trials involving refractory UC patients were systematically searched in electronic databases, including MEDLINE, EMBASE, and the Cochrane Library. Data were independently extracted by three investigators. Risk ratios (RRs) with 95% confidence intervals (CI) were calculated for key outcomes: remission, response, mucosal healing, and serious adverse events using random-effects models. Network meta-analysis, utilizing a frequency model, established comparative rankings, with surface under cumulative ranking curve (SUCRA) determining optimal treatments.

**Results:**

A total of nineteen studies, containing 5,450 patients, were included. In the induction phase, Qing-Chang-Hua-Shi emerged as the most effective drug for remission (RR vs placebo 0.78, 95% CI 0.64–0.95; SUCRA, 0.89). Cyclosporine showed the most promising effect for treatment response (RR 0.22, 95% CI 0.07–0.67; SUCRA, 0.90). Tacrolimus was most effective in terms of mucosal healing (RR 0.57, 95% CI 0.44–0.73; SUCRA, 0.89). Recombinant interferon-β-1a exhibited the lowest risk of serious adverse events (RR 0.08, 95% CI 0.01–0.62; SUCRA, 0.94).

**Conclusion:**

No single drug demonstrated consistent superiority across all four evaluated outcomes for refractory ulcerative colitis. Treatment strategies should therefore be individualized according to specific clinical objectives and the quality of available evidence.

## Introduction

Ulcerative colitis (UC) is a chronic inflammatory bowel disease characterized by inflammation and ulcers in the inner lining of the colon and rectum. In 2023, the global prevalence of UC was estimated to be five million cases, with a rising incidence globally [1]. Despite its prevalence, the pathogenesis of UC remains unclear, and it cannot be cured [2].

Conventional treatments for mild to severe UC encompass 5-aminosalicylates, corticosteroids, and immunosuppressive agents. Due to the varied pathogenesis of UC, there is a difference in treatment effect, and consequently, some UC becomes refractory. Refractory ulcerative colitis represents a severe and persistent form of UC that resists or fails to respond adequately to standard medical treatments, with an occurrence rate of 10%−20% in UC patients [1]. For patients grappling with resistance or intolerance to medications, some guidelines advocate contemplating colectomy [3]. Others recommend considering the use of infliximab or cyclosporine [4]. Given the substantial physical and psychosocial burden of colectomy [5], avoiding surgery through effective medical therapy is a central priority for both patients and clinicians. The emergence of biologics, small-molecule drugs and traditional Chinese medicine has introduced promising avenues for colitis treatment [6,7].

A multitude of drugs has been explored in patients with refractory UC, demonstrating varying degrees of effectiveness [8]. Prior studies have evaluated cyclosporine, tacrolimus, and infliximab in this population [9–11]. However, treatment outcomes in refractory UC are often unsatisfactory, and the disease is prone to relapse, underscoring the need for broad comparative evaluations of available therapies. To date, no large-scale and comprehensive analyses have directly compared conventional agents with newer therapies – including biologics, small-molecule drugs, and traditional Chinese medicines – in patients with refractory UC. Novel drugs may hold greater therapeutic potential owing to their targeted mechanisms of action and emerging clinical evidence. Moreover, most existing studies have primarily focused on the efficacy of clinical remission and response, while evidence on mucosal healing remains limited. These gaps in knowledge hinder precise clinical decision-making in the second-line management of refractory UC. To address this gap, our study scrutinized randomized controlled trials (RCTs) encompassing conventional drugs, novel biologics, small-molecule drugs, and Chinese herbal formula in patients with refractory UC. Through a network meta-analysis, we aimed to facilitate direct and indirect comparisons of efficacy and safety among different drugs, providing evidence-based references for physicians in the management of refractory UC.

## Methods

This study was conducted in accordance with the Preferred Reporting Items for Systematic Reviews and Meta-analyses (PRISMA) statement [12] (S1 File). The protocol was registered with the international prospective register of systematic reviews (PROSPERO CRD42023482185).

### Data sources and search strategy

We systematically searched MEDLINE, the Cochrane Library, and Embase. The search timeframe was set from database inception to January 31, 2025. According to the PICOS framework, “Refractory Ulcerative Colitis” “Ulcerative Colitis” and corresponding MeSH and Emtree subject terms were used to search for patients. Names of possible therapeutic drugs and “placebo” terms were employed to search for interventions and controls, while “randomized controlled trial” and corresponding MESH and EMTREE subject terms were used to search for study types. The specific search strategies are detailed in S2 File, Table S2.

### Study selection and outcomes assessment

Refractory UC is defined as the failure of glucocorticoids or 5-aminosalicylic acid [13,14]. Only RCTs involving adult patients with refractory UC were considered for inclusion to ensure the quality of evidence. The primary outcome was clinical remission, defined by the following criteria:

a. Total Mayo Score (TMS) of 2 or lower with no subscore exceeding 1, as assessed by the Mayo score.
b. Disease Activity Index (DAI) score of 2 or lower with no individual subscore higher than 1, as assessed by DAI.
c. Ulcerative Colitis Symptom Score (UCSS) of 2 or lower, accompanied by sigmoidoscopic remission as a Baron score of 0, as assessed by UCSS. in patients with refractory UC.

Secondary outcomes encompassed response to treatment, mucosal healing, and the occurrence of serious adverse events. Clinical response was defined as a decrease of at least 30% from baseline in Modified Mayo Score or TMS, along with a minimum 1-point decrease from baseline in rectal bleeding subscore, or a reduction of 4 points from DAI. mucosal healing was defined as Mayo endoscopic score or mucosal appearance subscore of DAI of 1 or lower. Serious adverse events were determined according to the Common Terminology Criteria for Adverse Events. There were no restrictions on the language of publication. RCTs were required report at least one of these four outcomes; otherwise, they would be excluded.

Two investigators (Y. Xu and W. Jiang) independently determined the inclusion and exclusion of articles by reviewing the titles and abstracts. A third researcher (M. Jiang) participated in article screening while reviewing the original article. Any discrepancies or uncertainties were resolved through discussion and consensus among the three researchers.

### Data extraction and quality assessment

All data were independently extracted into prepared tables by three researchers (Y. Xu, W. Jiang and M. Jiang). The extracted information included basic article details such as title, first author, year of publication, country of publication, etc.; study information such as clinical trial registration number, patient inclusion criteria, sample size of the intervention and control groups, age, male-to-female ratio, administration, and duration of the study, etc. Additionally, information on clinical outcomes including remission rate, response rate, mucosal healing rate, colectomy rate, incidence of serious adverse events and the outcome evaluation criteria, were recorded.

Two investigators (M. Jiang and W. Jiang) utilized the Risk of Bias 2 Tool to assess the quality of the included articles, considering five perspectives: the randomization process, deviations from intended interventions, missing outcome data, measurement of the outcome, and selection of the reported result [15]. Any inconsistencies were arbitrated by a third investigator (Y. Xu), who made the final decision.

### Data synthesis and statistical analysis

We utilized Stata (version 17) to conduct a frequency-based network meta-analysis. Initially, we generated network graphs to illustrate all comparisons. As network plots lacked closed loops, loop inconsistency tests were deemed unnecessary. Risk ratios and their 95% confidence intervals for each outcome were computed using random-effects models. Treatment strategies were ranked by calculating and plotting the surface under the cumulative ranking curve (SUCRA), where a higher SUCRA indicating a greater probability of being the best treatment option in simulations.

To ensure the robustness of findings, heterogeneity was assessed across the combined results. We then carried out sensitivity analyses, which involved systematically excluding each study to observe the impact on the overall results and to identify potential sources of heterogeneity. Funnel plots were generated for visual inspection to evaluate the publication bias of included studies. The reliability and quality of evidence was evaluated using the Grading of Recommendations, Assessment, Development and Evaluation (GRADE) methodology.

## Results

**Fig 1:**
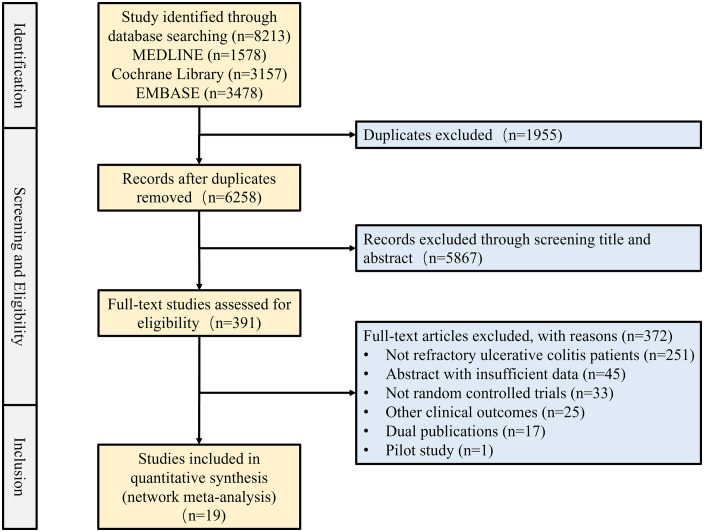
Process for article selection.

**Fig 2:**
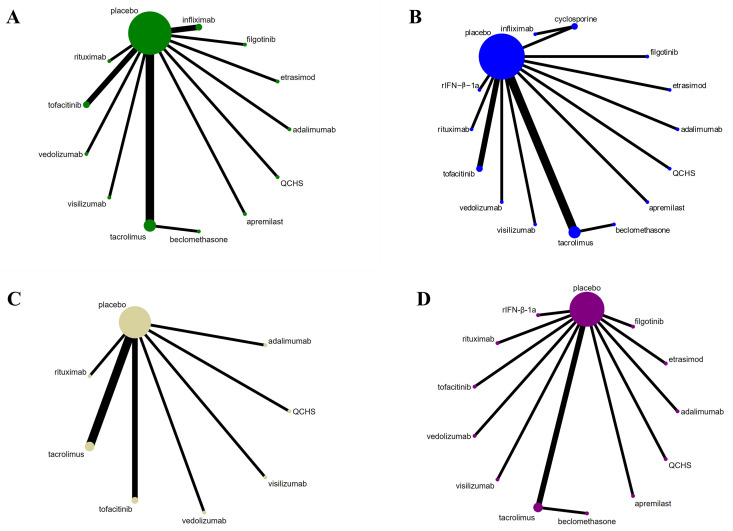
Network plots of comparisons on different outcomes of treatments in different groups for patients with refractory ulcerative colitis. **(A)** Comparison for remission. **(B)** Comparison for response. **(C)** Comparison for mucosal healing. **(D)** Comparison for serious adverse events.

The literature search yielded 8,213 original publications. A substantial portion of the literature was excluded for reasons such as studies not specifically targeting refractory cases. Ultimately, nineteen RCTs containing 5,450 patients were deemed eligible and included (Fig 1). Among these studies, 16 documented remissions during the induction phase [16–31], 17 reported responses to treatments [16–18, 20–24, 26–28, 30–35], 10 focused on mucosal healing [17, 20, 21, 23, 24, 26–28, 30, 31], and 13 detailed severe adverse events [16–18, 21–24, 26, 27, 29–31, 34]. For completeness, we provide the extracted raw data in S2 File, Table S3 and summarize the dosing regimens of all medications examined in S2 File, Table S4. Network diagrams illustrating the comparative treatments for these four outcomes are presented in Fig 2. Studies employed various evaluation systems such as the Mayo Score, the Lichtiger Score, Clinical Activity Score, Seo Index, and Disease Activity Index to assess drug effects in terms of clinical efficacy. The baseline characteristics of included literature are delineated in S2 File, Table S5.

### Efficacy in the induction phase

#### Clinical remission.

**Fig 3:**
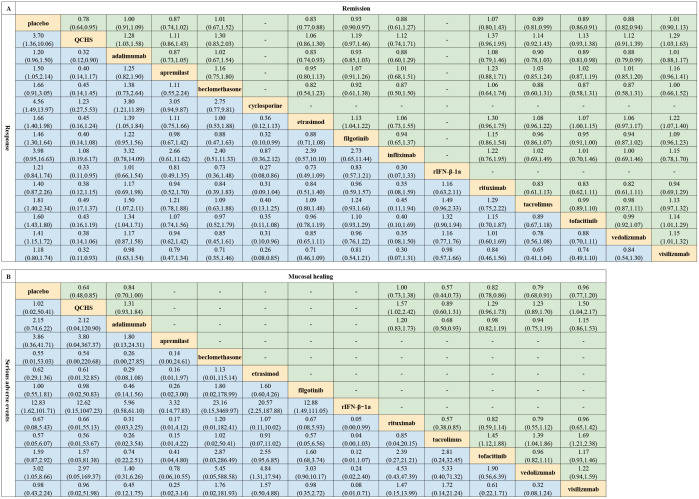
Pooled estimates of the network meta-analysis on outcomes. **(A)** Remission (on the upper triangle) and response (on the lower triangle). **(B)** Mucosal healing (on the upper triangle) and serious adverse events (on the lower triangle). Abbreviations: rIFN-β-1a, recombinant interferon-β-1a; QCHS, Qing-Chang-Hua-Shi.

**Fig 4:**
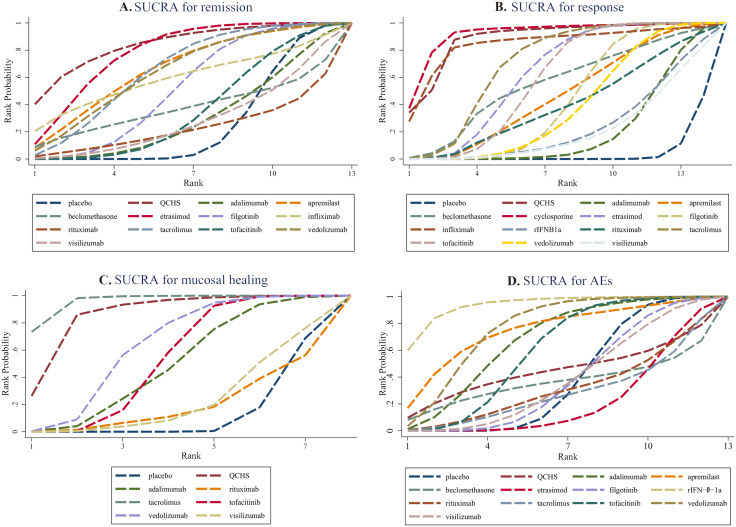
The cumulative ranking area (SUCRA) of treatment strategies based on probability of outcomes of (A) remission, (B)response, (C) mucosal healing, and (D)serious adverse event in the induction phase. Abbreviations: rIFN-β-1a, recombinant interferon-β-1a.

Data on remission effects in refractory UC were extracted from 5,183 patients in 16 clinical trials. As depicted in Fig 3A and S2 File, Figure S1, six of the twelve drugs achieved significantly higher remission rates compared with placebo, namely, Qing-Chang-Hua-Shi, etrasimod, filgotinib, tacrolimus, tofacitinib and vedolizumab. The effects were ranked according to SUCRA (Fig 4A), with Qing-Chang-Hua-Shi (SUCRA 88.5%) showing the highest probability of being the optimal treatment for achieving remission. SUCRA values for treatment strategies were presented in S2 File, Table S6.

#### Response.

Data on the response to treatments for refractory UC were derived from 5,360 patients in 17 clinical trials. Significant differences in responses were observed for eight drugs (Fig 3A and S2 File, Figure S2), namely, Qing-Chang-Hua-Shi, apremilast, cyclosporine, etrasimod, filgotinib, tacrolimus, tofacitinib, and vedolizumab. The effects were ranked according to SUCRA (Fig 4B), with cyclosporine (SUCRA 89.9%) having the greatest likelihood of the best therapeutic response.

#### Mucosal healing.

A limited number of RCTs reported mucosal healing outcomes, with data collected from 2,764 patients in 10 trials. Statistically significantly better mucosal healing rates were observed for four strategies (Fig 3B and S2 File, Figure S3), namely, Qing-Chang-Hua-Shi, tacrolimus, tofacitinib and vedolizumab. The effects were ranked according to SUCRA (Fig 4C), with tacrolimus (SUCRA 88.5%) having the highest probability of eliciting the best mucosal healing effect.

### Safety evaluation

Thirteen clinical trials reported the occurrence of serious adverse events in 4,659 patients. As shown in Fig 3B and S2 File, Figure S4, six of the twelve drugs had a lower risk of serious adverse events than placebo, with recombinant interferon-β-1a (rIFN-β-1a) (RR 0.08, 95% CI 0.01–0.62) and vedolizumab (RR 0.33, 95% CI 0.12–0.95) showed significantly lower risks of serious adverse events. The safety ranking (Fig 4D) demonstrated that rIFN-β-1a (SUCRA 93.8%) had the highest probability of being the therapeutic agent with the fewest serious adverse events, with vedolizumab (SUCRA 76.4%) and apremilast (SUCRA 74.9%) following in ranking.

### Heterogeneity assessment and sensitivity analysis

Some original publications involved multiple dose cohorts or diverse clinical trials, necessitating heterogeneity testing for the combined results of these treatments. As illustrated in S2 File, Figures S5–S8, heterogeneity assessment revealed low to moderate heterogeneity (0 ≤ I^2^ ≤ 50% and p > 0.05) among study groups. Only the combined datasets for etrasimod vs placebo (remission) and filgotinib vs placebo (response) showed a high degree of heterogeneity (S2 File, Figure S5C and S2 File, Figure S6D).

We performed sensitivity analyses by excluding each of the comparison groups with high heterogeneity (see S2 File, Figure S9). The results showed that dose may be one of the factors that influence patients’ clinical response to filgotinib.

### Risks of bias

The results of the article quality assessment are presented in S2 File, Figure S10, indicating minimal risk of bias overall. The results of publication bias, depicted funnel plots in S2 File, Figure S11, fell within acceptable limits, further reinforcing the robustness of our study.

### GRADE assessment

The GRADE assessment, detailed in S2 File, Table S7, evaluated the results for four outcomes, initially rated with high certainty due to all studies being randomized controlled trials. Among the 19 studies analyzed, one demonstrated a high overall risk of bias, necessitating a downgrade in certainty. Significant heterogeneity was noted between the two groups compared. Given the nature of network meta-analysis, the heterogeneity potentially impacts all network estimates. Sensitivity analyses were conducted to identify studies contributing to increased heterogeneity. These analyses showed some variation from the main findings, leading to a further downgrade in inconsistency. Additionally, certain comparisons were downgraded for imprecision. Ultimately, the certainty of the most treatment effects was classified as moderate to low.

## Discussion

Beyond the established medications like cyclosporine and infliximab, numerous clinical trials are actively challenging the landscape of refractory UC treatment. Given this scenario, we undertook a comprehensive systematic review and network meta-analysis, assimilating evidence from RCTs and undertaking direct and indirect comparisons of all potential therapeutic agents.

In terms of efficacy, although our analysis indicated that Qing-Chang-Hua-Shi may be effective in inducing remission during the induction phase, these findings should be viewed with caution due to the limited number of available studies of Qing-Chang-Hua-Shi and the relatively lower methodological quality compared to other included RCTs. In addition, etrasimod demonstrated superior clinical remission and favorable clinical response compared to several other treatments, though it may be associated with a higher incidence of adverse events such as anemia and headache. Outcomes related to mucosal healing were not reported in clinical trials. As etrasimod has already been approved in multiple countries and regions, further real-world studies are warranted to provide additional evidence regarding its effectiveness and safety profile.

In terms of safety, rIFN-β-1a emerges with the lowest risk of serious adverse events. This aspect underscores the potential of this biologic treatment as a safer alternative, especially for patients who may be at higher risk of complications from other more aggressive treatments. Despite this, there remains limited evidence for its efficacy, particularly in terms of remission and mucosal healing.

Taking into account efficacy and safety comprehensively, several therapeutic agents emerged as preferred treatment options for refractory ulcerative colitis. Vedolizumab appears to offer the best balance of efficacy and safety, demonstrating consistently good rankings in clinical remission, mucosal healing, and particularly strong safety outcomes. This finding aligns with current international guidelines, which recommend vedolizumab for patients with moderately to severely active ulcerative colitis who have an inadequate response or intolerance to conventional therapy [36,37]. Although vedolizumab is widely used as a step-up therapy in the management of ulcerative colitis, our findings add novelty by quantitatively positioning it among multiple advanced therapies within a unified evidence framework. This comparative synthesis, integrating both efficacy and safety outcomes, extends beyond prior pairwise or head-to-head analyses and provides a more comprehensive evaluation of its overall therapeutic value in refractory UC. In addition to vedolizumab, tacrolimus, notably effective in achieving mucosal healing, could be especially recommended for patients prioritizing endoscopic improvement, although its higher risk of adverse events necessitates careful monitoring. Etrasimod exhibited excellent efficacy in clinical remission and response; however, given its higher potential for anemia and headaches, caution should be exercised regarding its safety profile. rIFN-β-1a, while not showing strong clinical efficacy, provides superior safety, making it potentially beneficial in patients at heightened risk of serious adverse events. Conversely, despite their promising outcomes, treatments such as Qing-Chang-Hua-Shi warrant further high-quality randomized trials and real-world studies before routine clinical recommendations can be confidently made. Based on the findings of this study and previous evidence, the recommended therapeutic strategies for refractory ulcerative colitis are summarized in Table 1.

**Table 1:** Decision-making for drug selection in refractory UC.

Clinical scenario/ treatment target	Preferred option	Key considerations	Alternative option
Achieve clinical remission	Qing-Chang-Hua-Shi	Evidence base relatively small and lower methodological quality.	Etrasimod, tacrolimus, vedolizumab,
Achieve significant clinical response	Cyclosporine	Requires therapeutic drug monitoring and renal function checks.	Infliximab, Qing-Chang-Hua-Shi, tacrolimus
Prioritize mucosal healing	Tacrolimus	Monitor nephrotoxicity and neurotoxicity.	Qing-Chang-Hua-Shi, vedolizumab, tofacitinib
Prioritize safety	Recombinant IFN-β-1a	Limited efficacy data for remission.	Apremilast, vedolizumab, adalimumab
Overall balance	Vedolizumab	Consistently favorable across remission, mucosal healing, and safety; relatively high quality of evidence.	Qing-Chang-Hua-Shi

Our findings are aligned with previous research [9–11,38,39] but go further by including a wider array of therapeutic options, including newer biologics and small-molecule drugs that have been shown to be effective in other inflammatory conditions. For instance, our analysis supports existing evidence that tacrolimus and cyclosporine are effective in managing refractory cases [10], but it also suggests that these drugs do not consistently achieve clinical remission as effectively as some newer treatments [9].

Our study introduces a new outcome, mucosal healing, for which limited evidence-based studies related to ulcerative UC have been reported in the past. Research has proved that endoscopic mucosal healing is a pivotal prognostic parameter in the treatment of patients with UC [40]. Mucosal healing can serve as a predictor for long-term clinical remission, steroid-free remission, and colectomy-free survival in patients with UC [41]. Given its importance, conducting a network meta-analysis of mucosal healing endpoints becomes imperative. Our findings suggest that tacrolimus is particularly effective in inducing mucosal healing, providing a basis for their preferential use in clinical practice. In contrast, biologics, such as adalimumab, rituximab, and visilizumab, demonstrate limited effectiveness in this specific endpoint. Their performance in other endpoints appears similar, leading to the conclusion that, under conditions where alternative options are available, these biologics may not be the most suitable for the treatment of UC.

In this investigation, we did not analyze the outcome of colectomy. While we collected colectomy rates during information extraction from the literature, only one clinical trial, in addition to those previously reported, provided colectomy endpoints. The results indicated a higher risk of colectomy in the visilizumab group compared to the control group [26]. Given this circumstance, a new analysis of colectomy was not conducted.

Several strengths enhance the credibility and utility of our study. Firstly, the inclusion criteria were stringent, with only randomized controlled trials (RCTs) considered, minimizing the risk of bias. Secondly, the comprehensive nature of the data analysis, including a detailed heterogeneity analysis, sensitivity analysis, evaluation of bias, and GRADE assessment, ensures that the findings are reliable. Furthermore, by examining multiple outcomes, including three efficacy measures and one safety outcome, our study provides a holistic view of the treatment landscape, offering valuable guidance for clinical decision-making.

However, our study also has limitations. First, Due to incomplete documentation in primary literature, not every treatment reported all four outcomes. In total, 14 drugs were included, allowing for comparisons on the response outcome for all drugs. However, for the remission outcome, only 12 drugs were compared, excluding cyclosporine and rIFN-β-1a. Similarly, for serious adverse event outcomes, 12 drugs were compared, excluding cyclosporine and infliximab. Mucosal healing outcomes were reported by even fewer trials, with only seven drugs compared, excluding apremilast, beclomethasone, cyclosporine, etrasimod, filgotinib, infliximab, and rIFN-β-1a. Second, although our network included multiple interventions, the network plots lacked closed loops. This structure limited the ability to evaluate consistency between direct and indirect comparisons, which is an inherent limitation of the present analysis. Additionally, while our study included a diverse range of pharmacological treatments, the variability in the drugs’ mechanisms of action and the complexity of refractory ulcerative colitis pathology mean that further research is needed to fully understand how these treatments can be best utilized in clinical practice.

## Conclusion

In conclusion, no single treatment showed consistent superiority across all outcomes for refractory UC. Vedolizumab and tofacitinib offer balanced efficacy and safety, while tacrolimus is effective for mucosal healing but requires safety monitoring. Etrasimod shows promise but may cause more adverse events. rIFN-β-1a is safer but lacks strong efficacy data. Traditional medicines like Qing-Chang-Hua-Shi need further high-quality evidence. Treatment decisions should be guided by clinical goals and evidence quality, and future real-world studies are needed to support optimal therapeutic choices.

## Supporting information

S1 File PRISMA 2020 checklist. (DOCX)

S2 File Supplementary Materials. (DOCX)
