# Supplementary material for: Second-line treatment strategies of ulcerative colitis after conventional therapy failure: A systematic review and network meta-analysis of randomized controlled trials
Source: PLoS One. 2025 Dec 1;20(12):e0337222. doi: 10.1371/journal.pone.0337222 (PMC12668551; doi:10.1371/journal.pone.0337222)
Supplement: S2 File — (DOCX) [file pone.0337222.s002.docx]

**Supplementary Materials**

catalog

**Table S1.** PRISMA checklist**2**

**Table S2.** Search strategies**7**

**Table S3.** Extracted data of four outcomes **8**

**Table S4.** Administration of each treatment in clinical trials **10**

**Table S5.** Baseline characteristic of refractory UC patients **11**

**Table S6.** SURCA results in the induction phase **14**

**Table S7.** GRADE assessment **15**

**Figure S1.** Forest plot for pairwise comparisons of clinical remission **24**

**Figure S2.** Forest plot for pairwise comparisons of clinical response **25**

**Figure S3.** Forest plot for pairwise comparisons of mucosal healing **26**

**Figure S4.** Forest plot for pairwise comparisons of serious AEs**27**

**Figure S5.** Forest plots and heterogeneity of head-to-head comparisons for clinical remission **28**

**Figure S6.** Forest plots and heterogeneity of head-to-head comparisons for clinical response **30**

**Figure S7.** Forest plots and heterogeneity of head-to-head comparisons for mucosal healing **32**

**Figure S8.** Forest plots and heterogeneity of head-to-head comparisons for serious AEs **33**

**Figure S9.** Sensitivity analysis of filgotinib **34**

**Figure S10.** Risk of bias assessment **35**

**Figure S11.** Funnel plots**36**

**References** **38**

**Supplementary Table 1 Checklist of the PRISMA extension for network meta-analysis**

| **Section and Topic** | **Item #** | **Checklist item** | **Location where item is reported** |
| --- | --- | --- | --- |
| **TITLE** | | |  |
| Title | 1 | Identify the report as a systematic review. | Title p1 |
| **ABSTRACT** | | |  |
| Abstract | 2 | See the PRISMA 2020 for Abstracts checklist. | Abstract p2 |
| **INTRODUCTION** | | |  |
| Rationale | 3 | Describe the rationale for the review in the context of existing knowledge. | Paragraph 1-4 of “introduction” section p3 |
| Objectives | 4 | Provide an explicit statement of the objective(s) or question(s) the review addresses. | Paragraph 1 of the “Methods” p4 |
| **METHODS** | | |  |
| Eligibility criteria | 5 | Specify the inclusion and exclusion criteria for the review and how studies were grouped for the syntheses. | “Study selection and outcomes assessment” section p4-5 |
| Information sources | 6 | Specify all databases, registers, websites, organisations, reference lists and other sources searched or consulted to identify studies. Specify the date when each source was last searched or consulted. | “Data sources and search strategy” section p4 |
| Search strategy | 7 | Present the full search strategies for all databases, registers and websites, including any filters and limits used. | Suppl. P7 |
| Selection process | 8 | Specify the methods used to decide whether a study met the inclusion criteria of the review, including how many reviewers screened each record and each report retrieved, whether they worked independently, and if applicable, details of automation tools used in the process. | “Study selection and outcomes assessment” section p4-5 |
| Data collection process | 9 | Specify the methods used to collect data from reports, including how many reviewers collected data from each report, whether they worked independently, any processes for obtaining or confirming data from study investigators, and if applicable, details of automation tools used in the process. | Paragraph 3 of the “Study selection and outcomes assessment” p5 |
| Data items | 10a | List and define all outcomes for which data were sought. Specify whether all results that were compatible with each outcome domain in each study were sought (e.g. for all measures, time points, analyses), and if not, the methods used to decide which results to collect. | “Study selection and outcomes assessment” p5 |
|  | 10b | List and define all other variables for which data were sought (e.g. participant and intervention characteristics, funding sources). Describe any assumptions made about any missing or unclear information. | “Data extraction and quality assessment” section p5-6 |
| Study risk of bias assessment | 11 | Specify the methods used to assess risk of bias in the included studies, including details of the tool(s) used, how many reviewers assessed each study and whether they worked independently, and if applicable, details of automation tools used in the process. | Paragraph 2 of “Data extraction and quality assessment” p6 |
| Effect measures | 12 | Specify for each outcome the effect measure(s) (e.g. risk ratio, mean difference) used in the synthesis or presentation of results. | “Data synthesis and statistical analysis” section p6 |
| Synthesis methods | 13a | Describe the processes used to decide which studies were eligible for each synthesis (e.g. tabulating the study intervention characteristics and comparing against the planned groups for each synthesis (item #5)). | Paragraph 2 of “Data extraction and quality assessment” p6 |
|  | 13b | Describe any methods required to prepare the data for presentation or synthesis, such as handling of missing summary statistics, or data conversions. | “Data synthesis and statistical analysis” section p6 |
|  | 13c | Describe any methods used to tabulate or visually display results of individual studies and syntheses. | “Data synthesis and statistical analysis” section p6 |
|  | 13d | Describe any methods used to synthesize results and provide a rationale for the choice(s). If meta-analysis was performed, describe the model(s), method(s) to identify the presence and extent of statistical heterogeneity, and software package(s) used. | “Data synthesis and statistical analysis” section p6 |
|  | 13e | Describe any methods used to explore possible causes of heterogeneity among study results (e.g. subgroup analysis, meta-regression). | Paragraph 2 of “Data synthesis and statistical analysis” section p6 |
|  | 13f | Describe any sensitivity analyses conducted to assess robustness of the synthesized results. | Paragraph 2 of “Data synthesis and statistical analysis” section p6 |
| Reporting bias assessment | 14 | Describe any methods used to assess risk of bias due to missing results in a synthesis (arising from reporting biases). | “Data synthesis and statistical analysis” section p6 |
| Certainty assessment | 15 | Describe any methods used to assess certainty (or confidence) in the body of evidence for an outcome. | “Data synthesis and statistical analysis” section p6 |
| **RESULTS** | | |  |
| Study selection | 16a | Describe the results of the search and selection process, from the number of records identified in the search to the number of studies included in the review, ideally using a flow diagram. | Paragraph 1 of “Results” p6-7, Figure 1 |
|  | 16b | Cite studies that might appear to meet the inclusion criteria, but which were excluded, and explain why they were excluded. | Paragraph 1 of “Results” p6-7, Figure 1 |
| Study characteristics | 17 | Cite each included study and present its characteristics. | Paragraph 1 of “Results” p6-7, Table S4 |
| Risk of bias in studies | 18 | Present assessments of risk of bias for each included study. | Suppl. p15-23, p35 |
| Results of individual studies | 19 | For all outcomes, present, for each study: (a) summary statistics for each group (where appropriate) and (b) an effect estimate and its precision (e.g. confidence/credible interval), ideally using structured tables or plots. | “Efficacy in the Induction Phase” and “Safety Evaluation” sections p7-8, Figure 3, Suppl. p24-p27 |
| Results of syntheses | 20a | For each synthesis, briefly summarise the characteristics and risk of bias among contributing studies. | Figure 3 and Figure 4 |
|  | 20b | Present results of all statistical syntheses conducted. If meta-analysis was done, present for each the summary estimate and its precision (e.g. confidence/credible interval) and measures of statistical heterogeneity. If comparing groups, describe the direction of the effect. | Figure 3 and Figure 4 |
|  | 20c | Present results of all investigations of possible causes of heterogeneity among study results. | S5-8 Figure |
|  | 20d | Present results of all sensitivity analyses conducted to assess the robustness of the synthesized results. | S9 Figure |
| Reporting biases | 21 | Present assessments of risk of bias due to missing results (arising from reporting biases) for each synthesis assessed. | “Risks of Bias” section, suppl. p35-p37 |
| Certainty of evidence | 22 | Present assessments of certainty (or confidence) in the body of evidence for each outcome assessed. | Figure 3 and Figure 4 |
| **DISCUSSION** | | |  |
| Discussion | 23a | Provide a general interpretation of the results in the context of other evidence. | Paragraph 1 and 2 of “Discussion” p9 |
|  | 23b | Discuss any limitations of the evidence included in the review. | Last paragraph of “Discussion” p12-13 |
|  | 23c | Discuss any limitations of the review processes used. | Last paragraph of “Discussion” p12-13 |
|  | 23d | Discuss implications of the results for practice, policy, and future research. | Last paragraph of “Discussion” p12-13 |
| **OTHER INFORMATION** | | |  |
| Registration and protocol | 24a | Provide registration information for the review, including register name and registration number, or state that the review was not registered. | Paragraph 1 of “Method” p4 |
|  | 24b | Indicate where the review protocol can be accessed, or state that a protocol was not prepared. | We create our own review protocols |
|  | 24c | Describe and explain any amendments to information provided at registration or in the protocol. | N |
| Support | 25 | Describe sources of financial or non-financial support for the review, and the role of the funders or sponsors in the review. | This work was supported by National Key Research and Development Program of China [grant number 2020YFC2008305] |
| Competing interests | 26 | Declare any competing interests of review authors. | No conflict of interest between authors |
| Availability of data, code and other materials | 27 | Report which of the following are publicly available and where they can be found: template data collection forms; data extracted from included studies; data used for all analyses; analytic code; any other materials used in the review. | All relevant data are within the manuscript and its additional file. The data are available from the corresponding author on reasonable request. |

† Text in italics indicates wording specific to reporting of network meta-analyses that has been added to guidance from the PRISMA statement.

‡ Abbreviations: PRISMA, Preferred Reporting Items for Systematic Reviews and Meta-Analysis; PICOS, population, intervention, comparators, outcomes, study design.

**Supplementary Table 2 Search strategy^*^**

| **Database** | MEDLINE |
| --- | --- |
| **P: patient** | **#1** (Refractory Ulcerative Colitis[Title/Abstract]) OR (Refractory Colitis[Title/Abstract]) OR (Colitis, Ulcerative[Title/Abstract]) OR (Idiopathic Proctocolitis[Title/Abstract]) OR (Ulcerative Colitis[Title/Abstract]) OR (Colitis Gravis[Title/Abstract]) OR (Inflammatory Bowel Disease, Ulcerative Colitis Type[Title/Abstract]) OR (Refractory Colitis[Title/Abstract]) OR (Colitis, Ulcerative[MeSH Terms]) |
| **I/C: intervention and control** | **#2 (**Tacrolimus[Title/Abstract]) OR (Prograf[Title/Abstract])OR (Prograft[Title/Abstract]) OR (FR 900506[Title/Abstract]) OR (FK 506[Title/Abstract]) OR (Tacrolimus[MeSH Terms]) |
|  | **#3** (Infliximab[Title/Abstract]) OR (Monoclonal Antibody cA2[Title/Abstract]) OR (Antibody cA2, Monoclonal[Title/Abstract]) OR (Inflectra[Title/Abstract]) OR (Remicade[Title/Abstract]) OR (Renflexis[Title/Abstract]) OR (Infliximab[MeSH Terms]) |
|  | **#4** (adalimumab[Title/Abstract]) OR (Humira[Title/Abstract]) OR (Amjevita[Title/Abstract]) OR (Cyltezo[Title/Abstract]) OR (D2E7 Antibody[Title/Abstract]) OR (adalimumab[MeSH Terms]) |
|  | **#5** (cyclosporine[Title/Abstract]) OR (Cyclosporine A[Title/Abstract]) OR (Cyclosporin A[Title/Abstract]) OR (Ciclosporin[Title/Abstract]) OR (Cyclosporin[Title/Abstract]) OR (cyclosporine[MeSH Terms]) |
|  | **#6** (Mesalazine[Title/Abstract]) OR (Mesalamine[Title/Abstract]) OR (5-Aminosalicylic Acid[Title/Abstract]) OR (Asacol[Title/Abstract]) OR (Asacolon[Title/Abstract]) OR (Ascolitin[Title/Abstract]) OR (Canasa[Title/Abstract]) OR (Claversal[Title/Abstract]) OR (Fivasa[Title/Abstract]) OR (Salofalk[Title/Abstract]) OR (Lixacol[Title/Abstract]) OR (Mesalamine Hydrochloride[Title/Abstract]) OR (Mesasal[Title/Abstract]) OR (Novo-5 ASA[Title/Abstract]) OR (Pentasa[Title/Abstract]) OR (Rowasa[Title/Abstract]) OR (5-Aminosalicylate[Title/Abstract]) OR (Mesalamine[MeSH Terms]) |
|  | **#7** (Glucocorticoids[Title/Abstract]) OR (Glucocorticoids[MeSH Terms]) OR (methylprednisolone[Title/Abstract]) OR (Metipred[Title/Abstract]) OR (Urbason[Title/Abstract]) OR (Medrol[Title/Abstract]) OR (Methylprednisolone[MeSH Terms]) OR (Prednisone[Title/Abstract]) OR (Dehydrocortisone[Title/Abstract]) OR (delta-Cortisone[Title/Abstract]) OR (Pronisone[Title/Abstract]) OR (Prednisone[MeSH Terms]) |
|  | **#8** (azathioprine[Title/Abstract]) OR (Azothioprine[Title/Abstract]) OR (Imurel[Title/Abstract]) OR (Imuran[Title/Abstract]) OR (Immuran[Title/Abstract]) OR (azathioprine[MeSH Terms]) |
|  | **#9** placebo OR placebos |
| **O: outcome** | **/** |
| **S: study type** | **#10** (randomized controlled trial[Publication Type]) OR (random[Title/Abstract]) OR (randomized[Title/Abstract]) OR (randomly[Title/Abstract]) OR (randomised[Title/Abstract]) OR (clinical trial[Publication Type]) NOT (review[Publication Type]) |
| **Total** | **#1 AND (#2 OR #3 OR #4 OR #5 OR #6 OR #7 OR #8 OR #9) AND #10** |

† The search strategies for Cochrane Library and EMBASE are similar to those of MEDLINE, with changes mainly in MESH terms and EMTREE terms.

**Supplementary Table 3 Extracted data of four outcomes**

| Study | Total number of experimental group | Total number of control group | Clinical remission rate | | Clinical response rate | | Mucosal healing rate | | Serious AEs | |
| --- | --- | --- | --- | --- | --- | --- | --- | --- | --- | --- |
|  |  |  | events of experimental group | events of control group | events of experimental group | events of control group | events of experimental group | events of control group | events of experimental group | events of control group |
| Danese 2020 | 57 | 58 | 18 | 7 | 35 | 27 | NA | NA | 0 | 2 |
|  | 55 |  | 12 |  | 37 |  |  |  | 1 |  |
| Feagan 2013 | 225 | 149 | 38 | 8 | 106 | 38 | 92 | 37 | 5 | 10 |
| Feagan 2021 | 277 | 137 | 53 | 21 | 164 | 64 | NA | NA | 50 | 13 |
|  | 245 |  | 64 |  | 163 |  |  |  |  |  |
|  | 285 | 142 | 27 | 6 | 102 | 25 |  |  |  |  |
|  | 262 |  | 30 |  | 139 |  |  |  |  |  |
| Jarnerot 2005 | 15 | 6 | 6 | 2 | NA | NA | NA | NA | NA | NA |
| Laharie 2012 | 58 | 57 | NA | NA | 50 | 48 | NA | NA | 9 | 14 |
| Lawrance 2017 | 11 | 10 | 5 | 0 | 8 | 1 | 8 | 1 | NA | NA |
| Leiper 2011 | 16 | 8 | 1 | 1 | 6 | 1 | 2 | 1 | 3 | 1 |
| Lichtiger 1994 | 11 | 9 | NA | NA | 9 | 0 | NA | NA | NA | NA |
| Lie 2020 | 43 | 42 | 16 | 15 | 27 | 25 | NA | NA | 0 | 0 |
| Musch 2005 | 32 | 29 | NA | NA | 18 | 10 | NA | NA | 1 | 6 |
|  | 30 |  |  |  | 11 |  |  |  | 0 |  |
| Ogata 2006 | 19 | 20 | 4 | 1 | 13 | 2 | 15 | 2 | 1 | 0 |
|  | 21 |  | 2 |  | 8 |  | 8 |  | 1 |  |
| Ogata 2012 | 32 | 30 | 3 | 0 | 16 | 4 | 14 | 4 | 0 | 0 |
| Probert 2003 | 23 | 20 | 9 | 6 | NA | NA | NA | NA | 0 | 2 |
| Sandborn 2010 | 84 | 43 | 7 | 4 | 46 | 20 | 24 | 11 | 14 | 7 |
| Sandborn 2017 | 476 | 122 | 88 | 10 | 285 | 40 | 149 | 19 | 16 | 5 |
|  | 429 | 112 | 71 | 4 | 236 | 32 | 122 | 13 | 18 | 9 |
| Sandborn 2022 | 465 | 124 | 53 | 1 | 237 | 29 | 103 | 8 | NA | NA |
| Sandborn 2023 | 274 | 135 | 74 | 10 | 171 | 46 | NA | NA | 20 | 9 |
|  | 222 | 112 | 55 | 17 | 138 | 46 |  |  | 6 | 2 |
| Shen 2021 | 60 | 59 | 19 | 7 | 56 | 43 | 26 | 9 | 0 | 0 |
| Suzuki 2014 | 87 | 96 | 12 | 11 | 37 | 34 | 34 | 29 | 2 | 7 |
|  | 90 |  | 9 |  | 45 |  | 40 |  | 4 |  |

**Supplementary Table 4 Administration of each treatment in clinical trials**

| Study | Drugs | Dosage | Frequency |
| --- | --- | --- | --- |
| Danese 2020 | Apremilast | 30 mg or 40 mg | Twice a day |
| Feagan 2013 | Vedolizumab | 300mg | Every 8 or 4 weeks |
| Feagan 2021 | Filgotinib | 200mg or 100mg | Once a day |
| Jarnerot2005 | Infliximab | 5mg/kg | Once |
| Laharie 2012 | Cyclosporin | 2mg/kg | Once a day |
| Laharie 2012 | Infliximab | 5mg/kg | On days 0, 14, and 12 |
| Lawrance 2017 | Tacrolimus | 0.5 mg/ml, 3ml | Twice a day |
| Leiper 2011 | Rituximab | 1g in 500ml of 0.9% saline | Twice, with an interval of 2 weeks between each |
| Lichtiger 1994 | Cyclosporine | 4mg/kg | Once a day |
| Lie 2020 | Tacrolimus | 2mg | Once a day |
| Lie 2020 | Beclomethasone | 3mg | Once a day |
| Musch 2005 | Recombinant interferon-β-1a (rIFN-β-1a) | 3MIU or 1MIU | 3 times a week |
| Ogata 2006 | Tacrolimus | 0.05mg/kg | Twice a day |
| Ogata 2012 | Tacrolimus | 1-2mg (calculated using through concentration) | Twice a day |
| Probert 2003 | Infliximab | 5mg/kg | At weeks 0 and 2 |
| Sandborn 2010 | Visilizumab | 5μg/kg | On day 1 and 2 |
| Sandborn 2017 | Tofacitinib | 5mg or 10mg | Twice a day |
| Sandborn 2022 | Tofacitinib | 5mg or 10mg | Twice a day |
| Sandborn 2023 | Etrasimod | 2mg | Once a day |
| Shen 2021 | Qing-Chang-Hua-Shi (QCHS) granule | 125g | Twice a day |
| Suzuki 2014 | Adalimumab | 80/40mg or 160/80mg | 80 mg at week 0, then 40 mg every other week or 160/80 mg at weeks 0/2, then 40 mg every other week |

**Supplementary Table 5 Baseline characteristic of included studies of patients with refractory ulcerative colitis**

| **Author, year** | **Refractory types** | **Country / Region** | **Intervention** | | | | **Controlled** | | | | **Efficacy evaluation** | **Assessment duration (week)** |
| --- | --- | --- | --- | --- | --- | --- | --- | --- | --- | --- | --- | --- |
|  |  |  | **Age** | **Male (%)** | **Mayo clinical score** | **Intervention** | **Age** | **Male (%)** | **Mayo clinical score** | **Controlled** |  |  |
| Danese 2020 ^1^ | Intolerant of, or had contraindications to one or more conventional UC therapies | 14 countries | 43.4±14.9 | 68 | 8.1±1.7 | Apremilast, high-dose | 43±14.0 | 57 | 8.2±1.7 | Placebo | Mayo score | 12 |
|  |  |  | 40±13.5 | 62 | 8.5±1.6 | Apremilast, low-dose |  |  |  |  |  |  |
| Feagan 2013 ^2^ | Unsuccessful previous treatment with one or more glucocorticoids, immunosuppressive medication, or TNF antagonists | 34 countries | 40±13.1 | 59 | 8.5±1.8 | Vedolizumab | 41±12.5 | 62 | 8.6±1.7 | Placebo | Mayo score | 6, 52 |
| Feagan 2021 ^3^ | Inadequate response to or intolerance to corticosteroids or immunosuppressants, naive to TNF antagonists | 40 countries | 42±13.1 | 50 | 8.6±1.3 | Filgotinib, high-dose | 41±12.9 | 64 | 8.7±1.3 | Placebo | Mayo score | 10, 58 |
|  |  |  | 42±13.3 | 57 | 8.6±1.4 | Filgotinib, low-dose |  |  |  |  |  |  |
|  | inadequate clinical response, loss of response to or intolerance to any TNF antagonist or vedolizumab |  | 43±14.2 | 57 | 9.2±1.4 | Filgotinib, high-dose | 44±14.9 | 61 | 9.3±1.4 | Placebo |  |  |
|  |  |  | 43±14.3 | 65 | 9.3±1.3 | Filgotinib, low-dose |  |  |  |  |  |  |
| Jarnerot 2005 ^4^ | Inadequate response to corticosteroids intravenously | Sweden and Denmark | 38 | 67 | NA | Infliximab | 36 | 38 | NA | Placebo | Seo index | 12 |
| Laharie 2012 ^5^ | Unsuccessful course of high-dose intravenous steroid therapy | France, Spain, Belgium, and Finland | 36  (26–52) | 53 | NA | Infliximab | 39  (26–50) | 52 | NA | Cyclosporine | Lichtiger score, Mayo score | 1, 14 |
| Lawrance 2017 ^6^ | Failure on or intolerant of conventional therapies | Australia | 48±4.9 | 73 | 8.6±0.4 | Tacrolimus | 39±4.8 | 40 | 9.6±0.5 | Placebo | Mayo score | 4 |
| Leiper 2011 ^7^ | Steroid-resistant | UK | 37±15 | 69 | 9.2±1.7 | Rituximab | 50±10 | 50 | 9.2±1.7 | Placebo | Mayo score | 4, 8, 12 |
| Lichtiger 1994 ^8^ | No response to introvenous corticosteroid | USA | 34 (18-60) | 36 | NA | Cyclosporine | 43 (20-65) | 56 | NA | Placebo | CAI | 2 |
| Lie 2020 ^9^ | Mesalamine-refractory or recurring UC | Netherlands and Belgium | 40  (18-75) | 37 | 7 (3–12) | Tacrolimus | 43  (19–76) | 33 | 7 (3–12) | Beclomethasone | Mayo score | 4 |
| Musch 2005 ^10^ | Steroid-refractory | Germany | 38 | 66 | 10 (median CAI) | rIFN-β-1a, high-dose | 38 | 52 | 10 (median CAI) | Placebo | CAI | 8 |
|  |  |  | 35 | 60 | 10 (median CAI) | rIFN-β-1a, low-dose |  |  |  |  |  |  |
| Ogata 2006 ^11^ | Unresponsiveness to oral or intravenous corticosteroid therapy | Japan | 33±10.3 | 47 | 9.2±1.2 (CAI) | Tacrolimus, high-level | 30±6.4 | 45 | 9.4±1.5 (CAI) | Placebo | DAI | 2 |
|  |  |  | 31±10.8 | 52 | 9.2±1.8 (CAI) | Tacrolimus, low-level |  |  |  |  |  |  |
| Ogata 2012 ^12^ | steroid-resistant or steroid-dependent | Japan | NA | NA | NA | Tacrolimus | NA | NA | NA | Placebo | DAI | 2 |
| Probert 2003 ^13^ | No response to conventional treatment with glucocorticoids | UK and Germany | 41 | NA | NA | Infliximab | 40 | NA | NA | Placebo | UC symptom score | 6 |
| Sandborn 2010 ^14^ | Failure on oral corticosteroid treatment | 14 countries | 40±12.9 | 52 | 10.7±0.9 | Visilizumab | 41±13.5 | 28 | 10.7±0.8 | Placebo | Mayo score | 6.4 |
| Sandborn 2017 ^15^ | Failure on or unacceptable side effects from at least one of the following agents: oral / intravenous glucocorticoids, azathioprine, mercaptopurine, infliximab, or adalimumab | North America and Europe | 41±14.1 | 58 | 9.0±1.4 | Tofacitinib | 42±15.3 | 63 | 9.1±1.4 | Placebo | Mayo score | 8, 52 |
|  |  |  | 41±13.5 | 60 | 9.0±1.5 | Tofacitinib | 40±13.2 | 49 | 8.9±1.5 | Placebo |  |  |
| Sandborn 2022 ^16^ | TNF inhibitors failure | Multinational | 41±14.2 | 59 | 9.2±1.4 | Tofacitinib | 40±14.7 | 52 | 9.1±1.5 | Placebo | Mayo score | 8, 52 |
| Sandborn 2023 ^17^ | Inadequate response, loss of response, or intolerance of at least one therapy approved for the treatment of ulcerative colitis | 40 countries | 41±14.0 | 53 | 9.0±1.5 | Etrasimod | 39±14.0 | 61 | 9.0±1.4 | Placebo | Mayo score | 12, 52 |
|  |  |  | 40±13.5 | 57 | 8.7±1.5 | Etrasimod | 40±13.3 | 63 | 8.8±1.5 | Placebo |  |  |
| Shen 2021 ^18^ | No response to 4 weeks of mesalazine therapy at the maximum dose | China | 44±11.8 | 53 | 8.04±0.93 | Qing-Chang-Hua-Shi | 40±11.36 | 63 | 7.85±0.94 | Placebo | Mayo score | 12 |
| Suzuki 2014 ^19^ | refractory to corticosteroids, immunomodulators, or both | Japan | 43±14.6 | 57 | 8.6±1.4 | Adalimumab high-dose | 41±13.6 | 73 | 8.5±1.6 | Placebo | Mayo score | 8, 32, 52 |
|  |  |  | 44±15.0 | 68 | 8.5±1.4 | Adalimumab low-dose |  |  |  |  |  |  |

† Abbreviations: CAI, clinical-activity index. DAI, disease activity index score. rIFN-β-1a, recombinant interferon-β-1a. TNF, tumor necrosis factor. UC, ulcerative colitis.

**Supplementary Table 6 SUCRA of network meta-analysis in the induction phase**

| **Treatments** | **Remission** | | |  | **Response** | | |  | **Mucosal healing** | | |  | **Serious adverse event** | | |
| --- | --- | --- | --- | --- | --- | --- | --- | --- | --- | --- | --- | --- | --- | --- | --- |
|  | **SUCRA (%)** | **PrBest (%)** | **Rank^*^** |  | **SUCRA (%)** | **PrBest (%)** | **Rank** |  | **SUCRA (%)** | **PrBest (%)** | **Rank** |  | **SUCRA (%)** | **PrBest (%)** | **Rank** |
| Placebo | 21.2 | 0 | 11 |  | 3.9 | 0 | 16 |  | 11.1 | 0 | 9 |  | 38.7 | 0 | 7 |
| QCHS | 88.5 | 47 | 1 |  | 85.0 | 23 | 3 |  | 78.0 | 12 | 3 |  | 44.7 | 10 | 6 |
| Adalimumab | 23.4 | 0 | 10 |  | 19.2 | 0 | 15 |  | 43.2 | 0 | 6 |  | 67.3 | 1 | 4 |
| Apremilast | 66.3 | 7 | 3 |  | 43.9 | 0 | 9 |  | / | / | / |  | 74.9 | 17 | 3 |
| Beclomethasone | 33.5 | 8 | 9 |  | 51.9 | 0 | 8 |  | / | / | / |  | 36.1 | 8 | 10 |
| Cyclosporine | / | / | / |  | 89.9 | 28 | 2 |  | / | / | / |  | / | / | / |
| Etrasimod | 85.0 | 12 | 2 |  | 58.3 | 0 | 6 |  | / | / | / |  | 21.6 | 0 | 13 |
| Filgotinib | 43.8 | 0 | 8 |  | 40.6 | 0 | 10 |  | / | / | / |  | 38.6 | 0 | 8 |
| Infliximab | 59.2 | 23 | 7 |  | 82.4 | 24 | 4 |  | / | / | / |  | / | / | / |
| rIFN-β-1a | / | / | / |  | 22.0 | 0 | 13 |  | / | / | / |  | 93.8 | 60 | 1 |
| Rituximab | 19.0 | 1 | 13 |  | 37.3 | 0 | 11 |  | 16.4 | 0 | 8 |  | 31.6 | 1 | 11 |
| Tacrolimus | 59.7 | 1 | 6 |  | 65.4 | 0 | 5 |  | 88.5 | 32 | 2 |  | 28.4 | 1 | 12 |
| Tofacitinib | 63.4 | 0 | 5 |  | 54.4 | 0 | 7 |  | 46.2 | 0 | 5 |  | 59.6 | 0 | 5 |
| Vedolizumab | 65.9 | 1 | 4 |  | 36.3 | 0 | 12 |  | 55.1 | 0 | 4 |  | 76.4 | 3 | 2 |
| Visilizumab | 21.1 | 0 | 12 |  | 20.0 | 0 | 14 |  | 19.8 | 0 | 7 |  | 38.4 | 0 | 9 |

*Ranked according to SUCRA.

Abbreviations: rIFN-β-1a, recombinant interferon-β-1a. SUCRA, surface under cumulative ranking. PrBest, probability of being the best treatment. QCHS, Qing-Chang-Hua-Shi.

**Supplementary Table 7 GRADE assessment**

| comparison | nature of the evidence | confidence | downgrading due to |
| --- | --- | --- | --- |
| remission | | | |
| QCHS vs placebo | direct | moderate | imprecision (-1) |
| adalimumab vs. placebo | direct | low | study limitation (-1), imprecision (-1) |
| apremilast vs. placebo | direct | moderate | study limitation (-1) |
| beclomethasone vs. placebo | indirect | low | indirectness (-1), imprecision (-1) |
| etrasimod vs. placebo | direct | high | none |
| filgotinib vs. placebo | direct | high | none |
| infliximab vs. placebo | indirect | low | indirectness (-1), imprecision (-1) |
| rituximab vs. placebo | direct | low | study limitation (-1), imprecision (-1) |
| tacrolimus vs. placebo | direct | moderate | inconsistency (-1) |
| tofacitinib vs. placebo | direct | high | none |
| vedolizumab vs. placebo | direct | high | none |
| visilizumab vs. placebo | direct | moderate | imprecision (-1) |
| adalimumab vs. QCHS | indirect | very low | indirectness (-1), inconsistency (-1), imprecision (-1) |
| apremilast vs. QCHS | indirect | low | indirectness (-1), imprecision (-1) |
| beclomethasone vs. QCHS | indirect | low | indirectness (-1), imprecision (-1) |
| etrasimod vs. QCHS | indirect | low | indirectness (-1), imprecision (-1) |
| filgotinib vs. QCHS | indirect | low | indirectness (-1), imprecision (-1) |
| infliximab vs. QCHS | indirect | low | indirectness (-1), imprecision (-1) |
| rituximab vs. QCHS | indirect | very low | study limitation (-1), indirectness (-1), imprecision (-1) |
| tacrolimus vs. QCHS | indirect | low | indirectness (-1), imprecision (-1) |
| tofacitinib vs. QCHS | indirect | low | indirectness (-1), imprecision (-1) |
| vedolizumab vs. QCHS | indirect | low | indirectness (-1), imprecision (-1) |
| visilizumab vs. QCHS | indirect | very low | indirectness (-1), inconsistency (-1), imprecision (-1) |
| apremilast vs. adalimumab | indirect | low | indirectness (-1), imprecision (-1) |
| beclomethasone vs. adalimumab | indirect | low | indirectness (-1), imprecision (-1) |
| etrasimod vs. adalimumab | indirect | low | indirectness (-1), inconsistency (-1), |
| filgotinib vs. adalimumab | indirect | low | indirectness (-1), imprecision (-1) |
| infliximab vs. adalimumab | indirect | low | indirectness (-1), imprecision (-1) |
| rituximab vs. adalimumab | indirect | very low | study limitation (-1), indirectness (-1), imprecision (-1) |
| tacrolimus vs. adalimumab | indirect | low | indirectness (-1), imprecision (-1) |
| tofacitinib vs. adalimumab | indirect | moderate | indirectness (-1) |
| vedolizumab vs. adalimumab | indirect | low | indirectness (-1), inconsistency (-1) |
| visilizumab vs. adalimumab | indirect | low | indirectness (-1), imprecision (-1) |
| beclomethasone vs. apremilast | indirect | low | indirectness (-1), imprecision (-1) |
| etrasimod vs. apremilast | indirect | low | indirectness (-1), imprecision (-1) |
| filgotinib vs. apremilast | indirect | low | indirectness (-1), imprecision (-1) |
| infliximab vs. apremilast | indirect | low | indirectness (-1), imprecision (-1) |
| rituximab vs. apremilast | indirect | very low | study limitation (-1), indirectness (-1), imprecision (-1) |
| tacrolimus vs. apremilast | indirect | low | indirectness (-1), imprecision (-1) |
| tofacitinib vs. apremilast | indirect | low | indirectness (-1), imprecision (-1) |
| vedolizumab vs. apremilast | indirect | low | indirectness (-1), imprecision (-1) |
| visilizumab vs. apremilast | indirect | low | indirectness (-1), imprecision (-1) |
| etrasimod vs. beclomethasone | indirect | low | indirectness (-1), imprecision (-1) |
| filgotinib vs. beclomethasone | indirect | low | indirectness (-1), imprecision (-1) |
| infliximab vs. beclomethasone | indirect | low | indirectness (-1), imprecision (-1) |
| rituximab vs. beclomethasone | indirect | very low | study limitation (-1), indirectness (-1), imprecision (-1) |
| tacrolimus vs. beclomethasone | direct | moderate | imprecision (-1) |
| tofacitinib vs. beclomethasone | indirect | low | indirectness (-1), imprecision (-1) |
| vedolizumab vs. beclomethasone | indirect | low | indirectness (-1), imprecision (-1) |
| visilizumab vs. beclomethasone | indirect | low | indirectness (-1), imprecision (-1) |
| filgotinib vs. etrasimod | indirect | very low | indirectness (-1), inconsistency (-1), imprecision (-1) |
| infliximab vs. etrasimod | indirect | low | indirectness (-1), imprecision (-1) |
| rIFN-β-1a vs. etrasimod | indirect | low | indirectness (-1), imprecision (-1) |
| rituximab vs. etrasimod | indirect | very low | study limitation (-1), indirectness (-1), imprecision (-1) |
| tacrolimus vs. etrasimod | indirect | low | indirectness (-1), imprecision (-1) |
| tofacitinib vs. etrasimod | indirect | very low | indirectness (-1), inconsistency (-1), imprecision (-1) |
| vedolizumab vs. etrasimod | indirect | very low | indirectness (-1), inconsistency (-1), imprecision (-1) |
| visilizumab vs. etrasimod | indirect | very low | indirectness (-1), inconsistency (-1), imprecision (-1) |
| infliximab vs. filgotinib | indirect | low | indirectness (-1), imprecision (-1) |
| rituximab vs. filgotinib | indirect | very low | study limitation (-1), indirectness (-1), imprecision (-1) |
| tacrolimus vs. filgotinib | indirect | low | indirectness (-1), imprecision (-1) |
| tofacitinib vs. filgotinib | indirect | low | indirectness (-1), inconsistency (-1), |
| vedolizumab vs. filgotinib | indirect | low | indirectness (-1), imprecision (-1) |
| visilizumab vs. filgotinib | indirect | low | ,indirectness (-1), imprecision (-1) |
| rituximab vs. infliximab | indirect | very low | study limitation (-1), indirectness (-1), imprecision (-1) |
| tacrolimus vs. infliximab | indirect | low | indirectness (-1), imprecision (-1) |
| tofacitinib vs. infliximab | indirect | moderate | indirectness (-1) |
| vedolizumab vs. infliximab | indirect | low | indirectness (-1), imprecision (-1) |
| visilizumab vs. infliximab | indirect | low | indirectness (-1), imprecision (-1) |
| tacrolimus vs. rituximab | indirect | very low | study limitation (-1), indirectness (-1), imprecision (-1) |
| tofacitinib vs. rituximab | indirect | very low | study limitation (-1), indirectness (-1), imprecision (-1) |
| vedolizumab vs. rituximab | indirect | very low | study limitation (-1), indirectness (-1), imprecision (-1) |
| visilizumab vs. rituximab | indirect | very low | study limitation (-1), indirectness (-1), imprecision (-1) |
| tofacitinib vs. tacrolimus | indirect | low | indirectness (-1), imprecision (-1) |
| vedolizumab vs. tacrolimus | indirect | low | indirectness (-1), imprecision (-1) |
| visilizumab vs. tacrolimus | indirect | low | indirectness (-1), imprecision (-1) |
| vedolizumab vs. tofacitinib | indirect | low | indirectness (-1), imprecision (-1) |
| visilizumab vs. tofacitinib | indirect | low | indirectness (-1), inconsistency (-1) |
| visilizumab vs. vedolizumab | indirect | very low | indirectness (-1), inconsistency (-1), imprecision (-1) |
| response | | | |
| QCHS vs placebo | direct | high | none |
| adalimumab vs. placebo | direct | low | study limitation (-1), imprecision (-1) |
| apremilast vs. placebo | direct | low | study limitation (-1), inconsistency (-1) |
| beclomethasone vs. placebo | indirect | low | indirectness (-1), imprecision (-1) |
| cyclosporine vs. placebo | direct | moderate | study limitation (-1) |
| etrasimod vs. placebo | direct | high | none |
| filgotinib vs. placebo | direct | moderate | inconsistency (-1) |
| infliximab vs. placebo | indirect | low | indirectness (-1), imprecision (-1) |
| rIFN-β-1a vs. placebo | direct | moderate | imprecision (-1) |
| rituximab vs. placebo | direct | low | study limitation (-1), imprecision (-1) |
| tacrolimus vs. placebo | direct | high | none |
| tofacitinib vs. placebo | direct | high | none |
| vedolizumab vs. placebo | direct | high | none |
| visilizumab vs. placebo | direct | moderate | imprecision (-1) |
| adalimumab vs. QCHS | indirect | low | indirectness (-1), inconsistency (-1) |
| apremilast vs. QCHS | indirect | low | indirectness (-1), imprecision (-1) |
| beclomethasone vs. QCHS | indirect | low | indirectness (-1), imprecision (-1) |
| cyclosporine vs. QCHS | indirect | low | indirectness (-1), imprecision (-1) |
| etrasimod vs. QCHS | indirect | low | indirectness (-1), imprecision (-1) |
| filgotinib vs. QCHS | indirect | low | indirectness (-1), imprecision (-1) |
| infliximab vs. QCHS | indirect | low | indirectness (-1), imprecision (-1) |
| rIFN-β-1a vs. QCHS | indirect | moderate | indirectness (-1) |
| rituximab vs. QCHS | indirect | very low | study limitation (-1), indirectness (-1), imprecision (-1) |
| tacrolimus vs. QCHS | indirect | low | indirectness (-1), imprecision (-1) |
| tofacitinib vs. QCHS | indirect | low | indirectness (-1), imprecision (-1) |
| vedolizumab vs. QCHS | indirect | low | indirectness (-1), imprecision (-1) |
| visilizumab vs. QCHS | indirect | low | indirectness (-1), inconsistency (-1) |
| apremilast vs. adalimumab | indirect | low | indirectness (-1), imprecision (-1) |
| beclomethasone vs. adalimumab | indirect | low | indirectness (-1), imprecision (-1) |
| cyclosporine vs. adalimumab | indirect | moderate | indirectness (-1) |
| etrasimod vs. adalimumab | indirect | low | indirectness (-1), inconsistency (-1) |
| filgotinib vs. adalimumab | indirect | low | indirectness (-1), imprecision (-1) |
| infliximab vs. adalimumab | indirect | low | indirectness (-1), imprecision (-1) |
| rIFN-β-1a vs. adalimumab | indirect | low | indirectness (-1), imprecision (-1) |
| rituximab vs. adalimumab | indirect | very low | study limitation (-1), indirectness (-1), imprecision (-1) |
| tacrolimus vs. adalimumab | indirect | moderate | indirectness (-1) |
| tofacitinib vs. adalimumab | indirect | low | indirectness (-1), inconsistency (-1) |
| vedolizumab vs. adalimumab | indirect | low | indirectness (-1), imprecision (-1) |
| visilizumab vs. adalimumab | indirect | low | indirectness (-1), imprecision (-1) |
| beclomethasone vs. apremilast | indirect | low | indirectness (-1), imprecision (-1) |
| cyclosporine vs. apremilast | indirect | low | indirectness (-1), imprecision (-1) |
| etrasimod vs. apremilast | indirect | low | indirectness (-1), imprecision (-1) |
| filgotinib vs. apremilast | indirect | low | ,indirectness (-1), imprecision (-1) |
| infliximab vs. apremilast | indirect | low | ,indirectness (-1), imprecision (-1) |
| rIFN-β-1a vs. apremilast | indirect | low | ,indirectness (-1), imprecision (-1) |
| rituximab vs. apremilast | indirect | very low | study limitation (-1), indirectness (-1), imprecision (-1) |
| tacrolimus vs. apremilast | indirect | low | indirectness (-1), imprecision (-1) |
| tofacitinib vs. apremilast | indirect | low | indirectness (-1), imprecision (-1) |
| vedolizumab vs. apremilast | indirect | low | indirectness (-1), imprecision (-1) |
| visilizumab vs. apremilast | indirect | low | indirectness (-1), imprecision (-1) |
| cyclosporine vs. beclomethasone | indirect | low | indirectness (-1), imprecision (-1) |
| etrasimod vs. beclomethasone | indirect | low | indirectness (-1), imprecision (-1) |
| filgotinib vs. beclomethasone | indirect | low | indirectness (-1), imprecision (-1) |
| infliximab vs. beclomethasone | indirect | low | indirectness (-1), imprecision (-1) |
| rIFN-β-1a vs. beclomethasone | indirect | low | indirectness (-1), imprecision (-1) |
| rituximab vs. beclomethasone | indirect | very low | study limitation (-1), indirectness (-1), imprecision (-1) |
| tacrolimus vs. beclomethasone | direct | moderate | imprecision (-1) |
| tofacitinib vs. beclomethasone | indirect | low | indirectness (-1), imprecision (-1) |
| vedolizumab vs. beclomethasone | indirect | low | indirectness (-1), imprecision (-1) |
| visilizumab vs. beclomethasone | indirect | low | indirectness (-1), imprecision (-1) |
| etrasimod vs. cyclosporine | indirect | low | indirectness (-1), imprecision (-1) |
| filgotinib vs. cyclosporine | indirect | low | indirectness (-1), inconsistency (-1) |
| infliximab vs. cyclosporine | direct | moderate | imprecision (-1) |
| rIFN-β-1a vs. cyclosporine | indirect | low | indirectness (-1), inconsistency (-1) |
| rituximab vs. cyclosporine | indirect | very low | study limitation (-1), indirectness (-1), imprecision (-1) |
| tacrolimus vs. cyclosporine | indirect | low | indirectness (-1), imprecision (-1) |
| tofacitinib vs. cyclosporine | indirect | low | indirectness (-1), imprecision (-1) |
| vedolizumab vs. cyclosporine | indirect | low | indirectness (-1), inconsistency (-1) |
| visilizumab vs. cyclosporine | indirect | moderate | indirectness (-1) |
| filgotinib vs. etrasimod | indirect | low | indirectness (-1), imprecision (-1) |
| infliximab vs. etrasimod | indirect | low | indirectness (-1), imprecision (-1) |
| rIFN-β-1a vs. etrasimod | indirect | low | indirectness (-1), imprecision (-1) |
| rituximab vs. etrasimod | indirect | very low | study limitation (-1), indirectness (-1), imprecision (-1) |
| tacrolimus vs. etrasimod | indirect | low | indirectness (-1), imprecision (-1) |
| tofacitinib vs. etrasimod | indirect | low | indirectness (-1), imprecision (-1) |
| vedolizumab vs. etrasimod | indirect | low | indirectness (-1), imprecision (-1) |
| visilizumab vs. etrasimod | indirect | low | indirectness (-1), imprecision (-1) |
| infliximab vs. filgotinib | indirect | low | indirectness (-1), imprecision (-1) |
| rIFN-β-1a vs. filgotinib | indirect | low | indirectness (-1), imprecision (-1) |
| rituximab vs. filgotinib | indirect | very low | study limitation (-1), indirectness (-1), imprecision (-1) |
| tacrolimus vs. filgotinib | indirect | low | indirectness (-1), imprecision (-1) |
| tofacitinib vs. filgotinib | indirect | low | indirectness (-1),imprecision (-1) |
| vedolizumab vs. filgotinib | indirect | low | indirectness (-1), imprecision (-1) |
| visilizumab vs. filgotinib | indirect | low | indirectness (-1), imprecision (-1) |
| rIFN-β-1a vs. infliximab | indirect | low | indirectness (-1), imprecision (-1) |
| rituximab vs. infliximab | indirect | very low | study limitation (-1), indirectness (-1), imprecision (-1) |
| tacrolimus vs. infliximab | indirect | low | indirectness (-1), imprecision (-1) |
| tofacitinib vs. infliximab | indirect | low | indirectness (-1), imprecision (-1) |
| vedolizumab vs. infliximab | indirect | low | indirectness (-1), imprecision (-1) |
| visilizumab vs. infliximab | indirect | low | indirectness (-1), imprecision (-1) |
| rituximab vs. rIFN-β-1a | indirect | very low | study limitation (-1), indirectness (-1), imprecision (-1) |
| tacrolimus vs. rIFN-β-1a | indirect | low | indirectness (-1), imprecision (-1) |
| tofacitinib vs. rIFN-β-1a | indirect | low | indirectness (-1), imprecision (-1) |
| vedolizumab vs. rIFN-β-1a | indirect | low | indirectness (-1), imprecision (-1) |
| visilizumab vs. rIFN-β-1a | indirect | low | indirectness (-1), imprecision (-1) |
| tacrolimus vs. rituximab | indirect | very low | study limitation (-1), indirectness (-1), imprecision (-1) |
| tofacitinib vs. rituximab | indirect | very low | study limitation (-1), indirectness (-1), imprecision (-1) |
| vedolizumab vs. rituximab | indirect | very low | study limitation (-1), indirectness (-1), imprecision (-1) |
| visilizumab vs. rituximab | indirect | very low | study limitation (-1), indirectness (-1), imprecision (-1) |
| tofacitinib vs. tacrolimus | indirect | low | indirectness (-1), imprecision (-1) |
| vedolizumab vs. tacrolimus | indirect | low | indirectness (-1), imprecision (-1) |
| visilizumab vs. tacrolimus | indirect | low | indirectness (-1), imprecision (-1) |
| vedolizumab vs. tofacitinib | indirect | low | indirectness (-1), imprecision (-1) |
| visilizumab vs. tofacitinib | indirect | low | indirectness (-1), imprecision (-1) |
| visilizumab vs. vedolizumab | indirect | low | indirectness (-1), imprecision (-1) |
| mucosal healing | | | |
| QCHS vs placebo | direct | high | none |
| adalimumab vs. placebo | direct | moderate | study limitation (-1) |
| rituximab vs. placebo | direct | moderate | study limitation (-1) |
| tacrolimus vs. placebo | direct | high | none |
| tofacitinib vs. placebo | direct | high | none |
| vedolizumab vs. placebo | direct | high | none |
| visilizumab vs. placebo | direct | moderate | imprecision (-1) |
| adalimumab vs. QCHS | indirect | low | indirectness (-1), imprecision (-1) |
| rituximab vs. QCHS | indirect | very low | study limitation (-1), indirectness (-1), inconsistency (-1), imprecision (-1) |
| tacrolimus vs. QCHS | indirect | low | indirectness (-1), imprecision (-1) |
| tofacitinib vs. QCHS | indirect | low | indirectness (-1), imprecision (-1) |
| vedolizumab vs. QCHS | indirect | moderate | indirectness (-1) |
| visilizumab vs. QCHS | indirect | low | indirectness (-1), inconsistency (-1) |
| rituximab vs. adalimumab | indirect | very low | study limitation (-1), indirectness (-1), imprecision (-1) |
| tacrolimus vs. adalimumab | indirect | low | indirectness (-1), inconsistency (-1) |
| tofacitinib vs. adalimumab | indirect | low | indirectness (-1), imprecision (-1) |
| vedolizumab vs. adalimumab | indirect | low | indirectness (-1), imprecision (-1) |
| visilizumab vs. adalimumab | indirect | low | indirectness (-1), imprecision (-1) |
| tacrolimus vs. rituximab | indirect | low | study limitation (-1), indirectness (-1) |
| tofacitinib vs. rituximab | indirect | very low | study limitation (-1), indirectness (-1), imprecision (-1) |
| vedolizumab vs. rituximab | indirect | very low | study limitation (-1), indirectness (-1), imprecision (-1) |
| visilizumab vs. rituximab | indirect | very low | study limitation (-1), indirectness (-1), I mprecision (-1) |
| tofacitinib vs. tacrolimus | indirect | moderate | indirectness (-1) |
| vedolizumab vs. tacrolimus | indirect | low | indirectness (-1), inconsistency (-1) |
| visilizumab vs. tacrolimus | indirect | moderate | indirectness (-1) |
| vedolizumab vs. tofacitinib | indirect | low | indirectness (-1), imprecision (-1) |
| visilizumab vs. tofacitinib | indirect | low | indirectness (-1), imprecision (-1) |
| visilizumab vs. vedolizumab | indirect | low | indirectness (-1), imprecision (-1) |
| AEs | | | |
| QCHS vs placebo | direct | moderate | imprecision (-1) |
| adalimumab vs. placebo | direct | low | study limitation (-1), imprecision (-1) |
| apremilast vs. placebo | direct | low | study limitation (-1), imprecision (-1) |
| beclomethasone vs. placebo | indirect | low | indirectness (-1), imprecision (-1) |
| etrasimod vs. placebo | direct | moderate | imprecision (-1) |
| filgotinib vs. placebo | direct | moderate | imprecision (-1) |
| rIFN-β-1a vs. placebo | direct | high | none |
| rituximab vs. placebo | direct | low | study limitation (-1), imprecision (-1) |
| tacrolimus vs. placebo | direct | moderate | imprecision (-1) |
| tofacitinib vs. placebo | direct | moderate | imprecision (-1) |
| vedolizumab vs. placebo | direct | high | none |
| visilizumab vs. placebo | direct | moderate | imprecision (-1) |
| adalimumab vs. QCHS | indirect | low | indirectness (-1), imprecision (-1) |
| apremilast vs. QCHS | indirect | low | indirectness (-1), imprecision (-1) |
| beclomethasone vs. QCHS | indirect | low | indirectness (-1), imprecision (-1) |
| etrasimod vs. QCHS | indirect | low | indirectness (-1), imprecision (-1) |
| filgotinib vs. QCHS | indirect | low | indirectness (-1), imprecision (-1) |
| rIFN-β-1a vs. QCHS | indirect | low | indirectness (-1), imprecision (-1) |
| rituximab vs. QCHS | indirect | very low | study limitation (-1), indirectness (-1), imprecision (-1) |
| tacrolimus vs. QCHS | indirect | low | indirectness (-1), imprecision (-1) |
| tofacitinib vs. QCHS | indirect | low | indirectness (-1), imprecision (-1) |
| vedolizumab vs. QCHS | indirect | low | indirectness (-1), imprecision (-1) |
| visilizumab vs. QCHS | indirect | low | indirectness (-1), imprecision (-1) |
| apremilast vs. adalimumab | indirect | low | indirectness (-1), imprecision (-1) |
| beclomethasone vs. adalimumab | indirect | low | indirectness (-1), imprecision (-1) |
| etrasimod vs. adalimumab | indirect | low | indirectness (-1), imprecision (-1) |
| filgotinib vs. adalimumab | indirect | low | indirectness (-1), imprecision (-1) |
| rIFN-β-1a vs. adalimumab | indirect | low | indirectness (-1), imprecision (-1) |
| rituximab vs. adalimumab | indirect | very low | study limitation (-1), indirectness (-1), imprecision (-1) |
| tacrolimus vs. adalimumab | indirect | low | indirectness (-1), imprecision (-1) |
| tofacitinib vs. adalimumab | indirect | low | indirectness (-1), imprecision (-1) |
| vedolizumab vs. adalimumab | indirect | low | indirectness (-1), imprecision (-1) |
| visilizumab vs. adalimumab | indirect | low | indirectness (-1), imprecision (-1) |
| beclomethasone vs. apremilast | indirect | low | indirectness (-1), imprecision (-1) |
| etrasimod vs. apremilast | indirect | low | indirectness (-1), imprecision (-1) |
| filgotinib vs. apremilast | indirect | low | indirectness (-1), imprecision (-1) |
| rIFN-β-1a vs. apremilast | indirect | low | indirectness (-1), imprecision (-1) |
| rituximab vs. apremilast | indirect | very low | study limitation (-1), indirectness (-1), imprecision (-1) |
| tacrolimus vs. apremilast | indirect | low | indirectness (-1), imprecision (-1) |
| tofacitinib vs. apremilast | indirect | low | indirectness (-1), imprecision (-1) |
| vedolizumab vs. apremilast | indirect | low | indirectness (-1), imprecision (-1) |
| visilizumab vs. apremilast | indirect | low | indirectness (-1), imprecision (-1) |
| etrasimod vs. beclomethasone | indirect | low | indirectness (-1), imprecision (-1) |
| filgotinib vs. beclomethasone | indirect | low | indirectness (-1), imprecision (-1) |
| rIFN-β-1a vs. beclomethasone | indirect | low | indirectness (-1), imprecision (-1) |
| rituximab vs. beclomethasone | indirect | very low | study limitation (-1), indirectness (-1), imprecision (-1) |
| tacrolimus vs. beclomethasone | direct | moderate | imprecision (-1) |
| tofacitinib vs. beclomethasone | indirect | low | indirectness (-1), imprecision (-1) |
| vedolizumab vs. beclomethasone | indirect | low | indirectness (-1), imprecision (-1) |
| visilizumab vs. beclomethasone | indirect | low | indirectness (-1), imprecision (-1) |
| filgotinib vs. etrasimod | indirect | low | indirectness (-1), imprecision (-1) |
| rIFN-β-1a vs. etrasimod | indirect | moderate | indirectness (-1) |
| rituximab vs. etrasimod | indirect | very low | study limitation (-1), indirectness (-1), imprecision (-1) |
| tacrolimus vs. etrasimod | indirect | low | indirectness (-1), imprecision (-1) |
| tofacitinib vs. etrasimod | indirect | low | indirectness (-1), imprecision (-1) |
| vedolizumab vs. etrasimod | indirect | moderate | indirectness (-1) |
| visilizumab vs. etrasimod | indirect | low | indirectness (-1), imprecision (-1) |
| rIFN-β-1a vs. filgotinib | indirect | moderate | indirectness (-1) |
| rituximab vs. filgotinib | indirect | very low | study limitation (-1), indirectness (-1), imprecision (-1) |
| tacrolimus vs. filgotinib | indirect | low | indirectness (-1), imprecision (-1) |
| tofacitinib vs. filgotinib | indirect | low | indirectness (-1), imprecision (-1) |
| vedolizumab vs. filgotinib | indirect | low | indirectness (-1), imprecision (-1) |
| visilizumab vs. filgotinib | indirect | low | indirectness (-1), imprecision (-1) |
| rituximab vs. rIFN-β-1a | indirect | low | study limitation (-1), indirectness (-1) |
| tacrolimus vs. rIFN-β-1a | indirect | low | indirectness (-1), imprecision (-1) |
| tofacitinib vs. rIFN-β-1a | indirect | low | indirectness (-1), imprecision (-1) |
| vedolizumab vs. rIFN-β-1a | indirect | low | indirectness (-1), imprecision (-1) |
| visilizumab vs. rIFN-β-1a | indirect | moderate | indirectness (-1) |
| tacrolimus vs. rituximab | indirect | very low | study limitation (-1), indirectness (-1), imprecision (-1) |
| tofacitinib vs. rituximab | indirect | very low | study limitation (-1), indirectness (-1), imprecision (-1) |
| vedolizumab vs. rituximab | indirect | very low | study limitation (-1), indirectness (-1), imprecision (-1) |
| visilizumab vs. rituximab | indirect | very low | study limitation (-1), indirectness (-1), imprecision (-1) |
| tofacitinib vs. tacrolimus | indirect | low | indirectness (-1), imprecision (-1) |
| vedolizumab vs. tacrolimus | indirect | low | indirectness (-1), imprecision (-1) |
| visilizumab vs. tacrolimus | indirect | low | indirectness (-1), imprecision (-1) |
| vedolizumab vs. tofacitinib | indirect | low | indirectness (-1), imprecision (-1) |
| visilizumab vs. tofacitinib | indirect | low | indirectness (-1), imprecision (-1) |
| visilizumab vs. vedolizumab | indirect | low | indirectness (-1), imprecision (-1) |

**
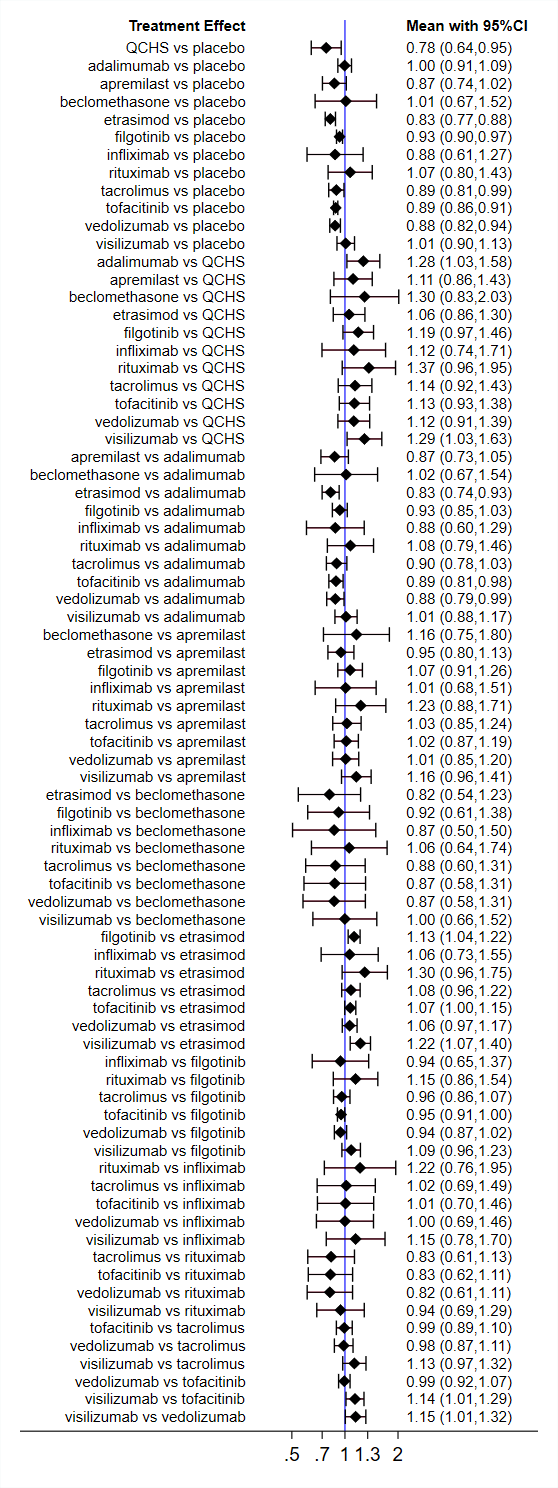
**

**Supplementary Figure 1 Forest plot for pairwise comparisons of clinical remission**

**
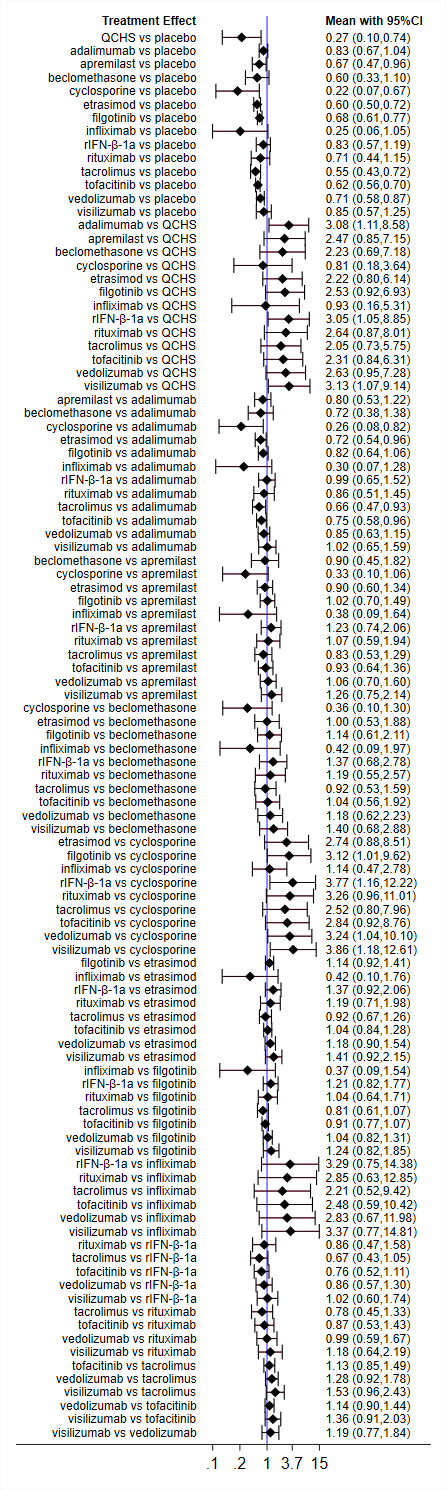
**

**Supplementary Figure 2 Forest plot for pairwise comparisons of clinical response**


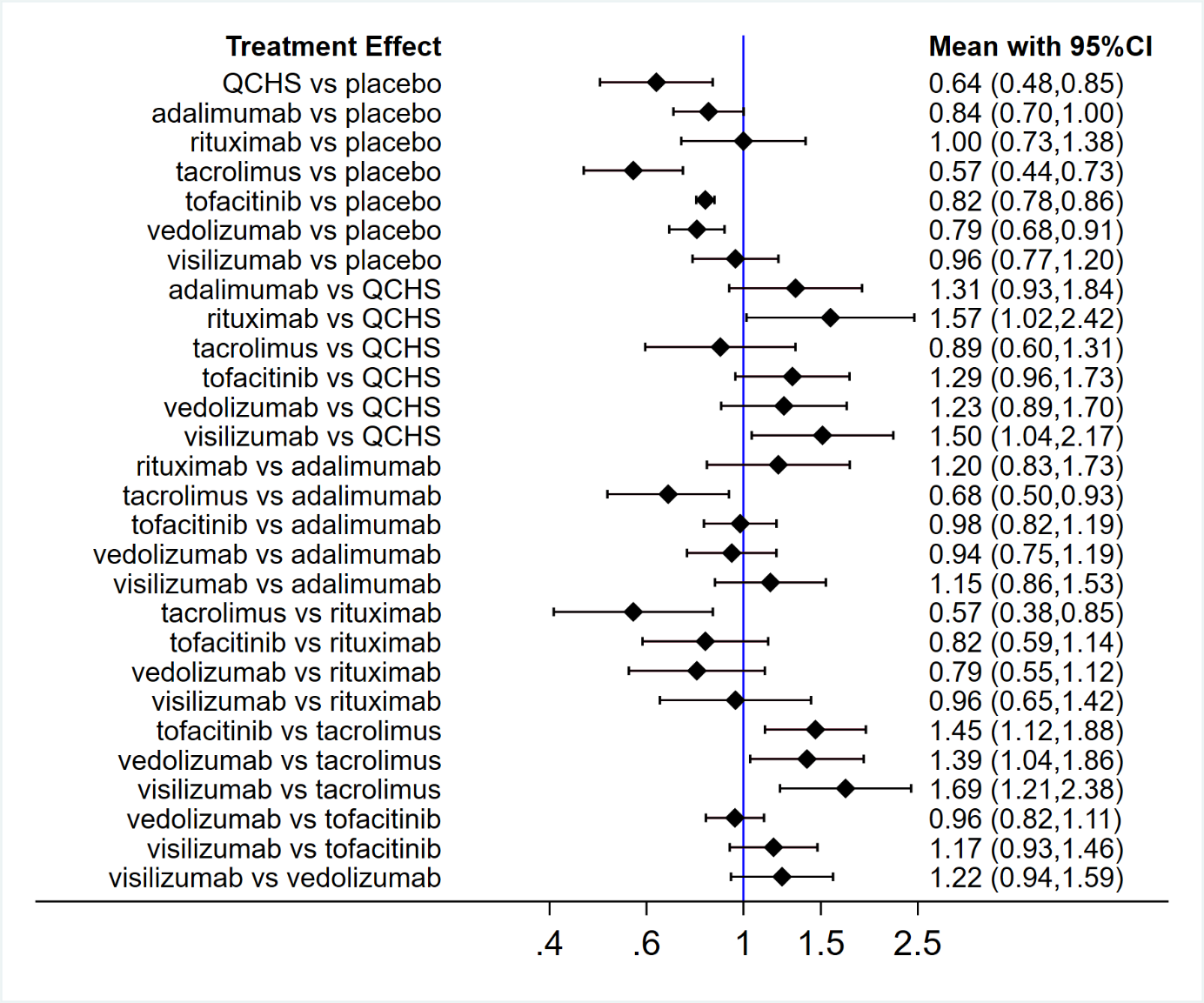


**Supplementary Figure 3 Forest plot for pairwise comparisons of mucosal healing**


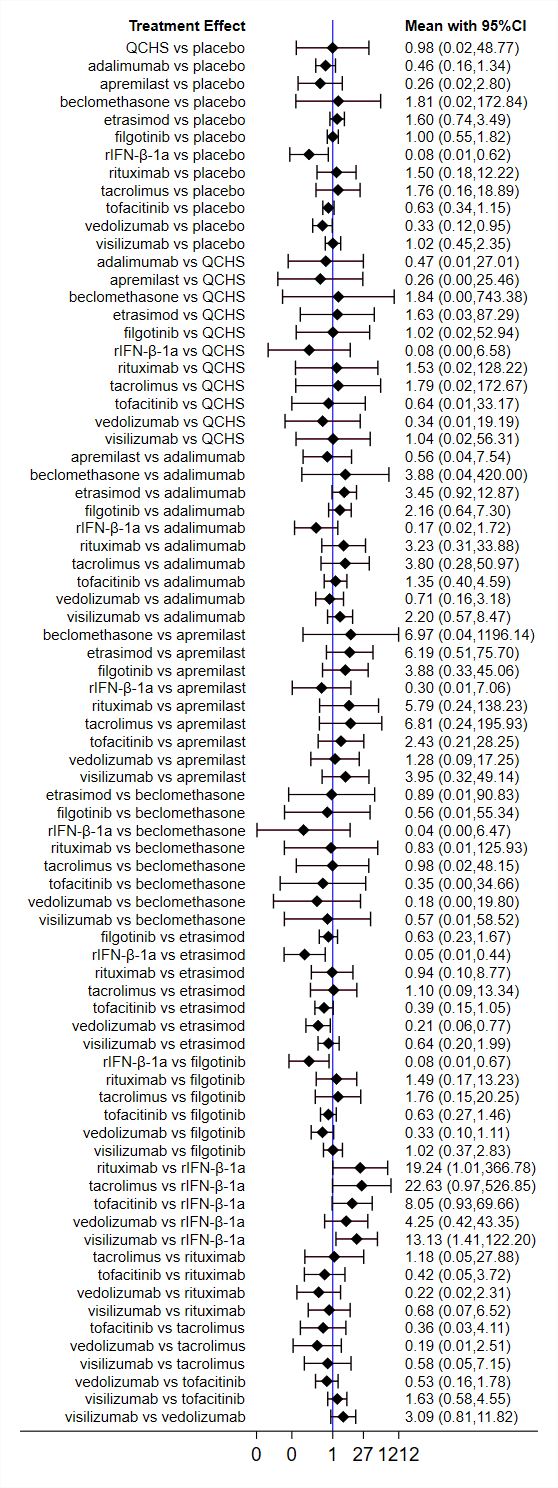


**Supplementary Figure 4 Forest plot for pairwise comparisons of serious adverse events**

**
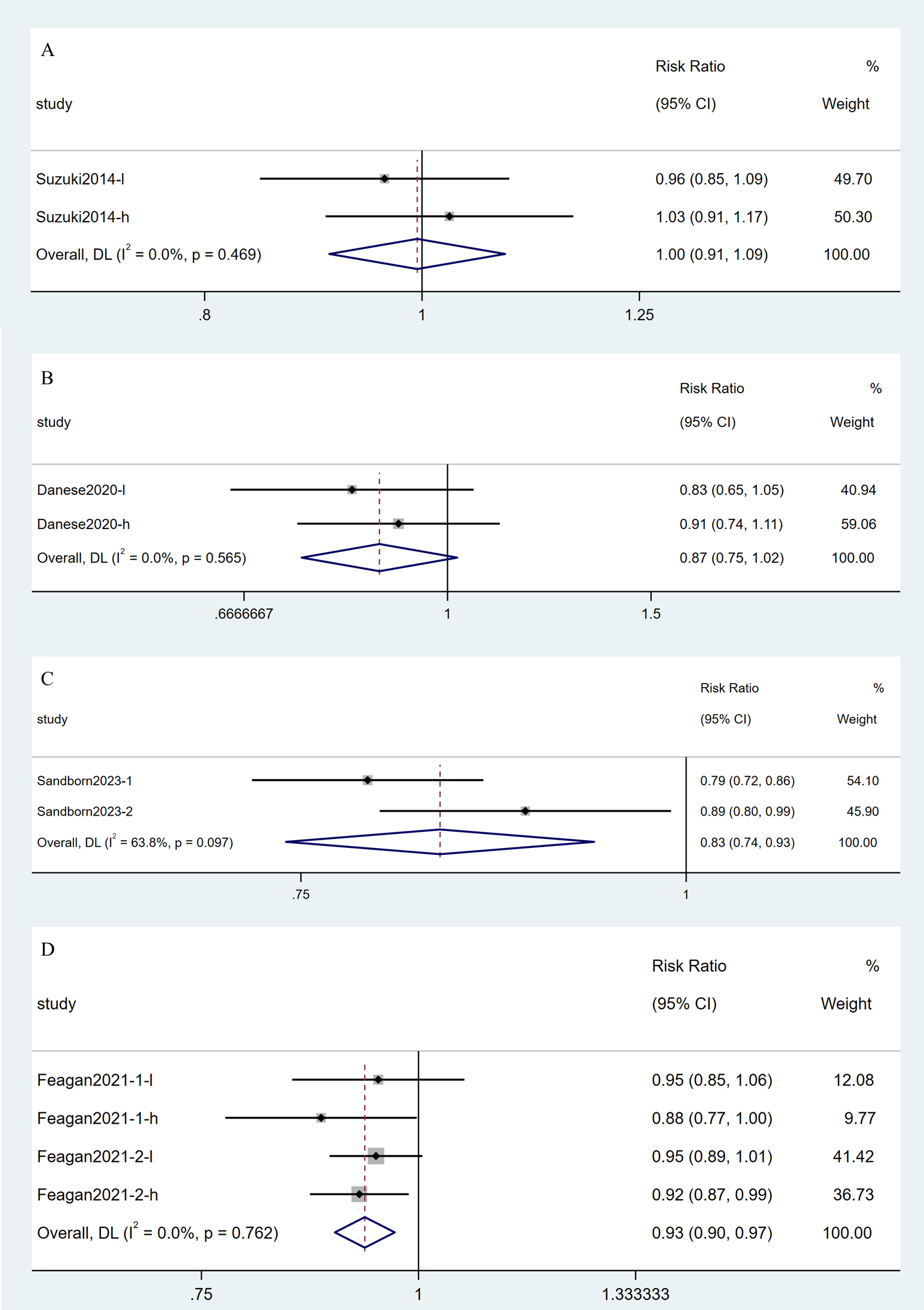

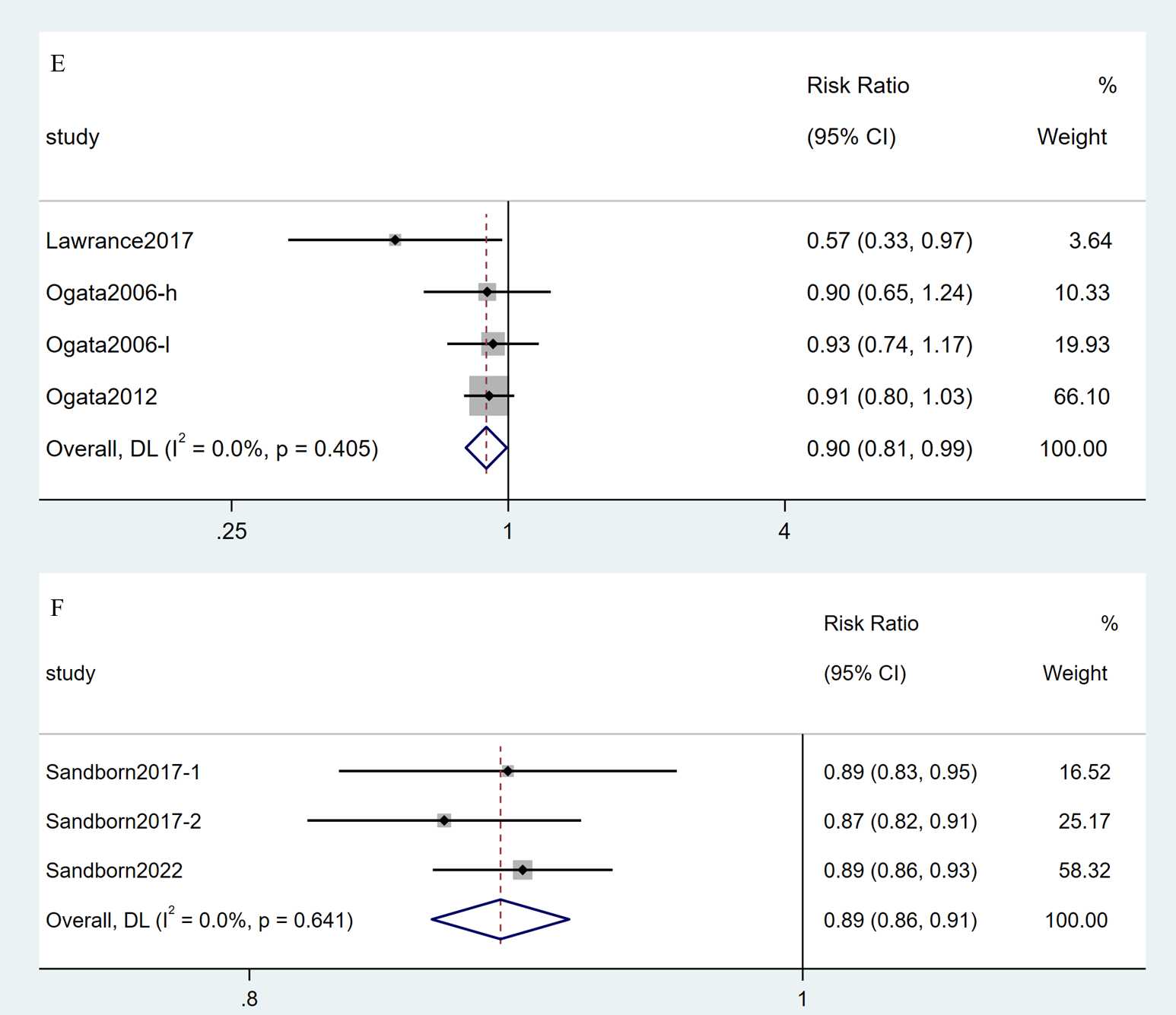
**

**Supplementary Figure 5 Forest plots and heterogeneity of head-to-head comparisons for clinical remission. (A) adalimumab vs placebo; (B) apremilast vs placebo; (C) etrasimod vs placebo; (D) filgotinib vs placebo; (E) tacrolimus vs placebo; (F) tofacitinib vs placebo.**

**
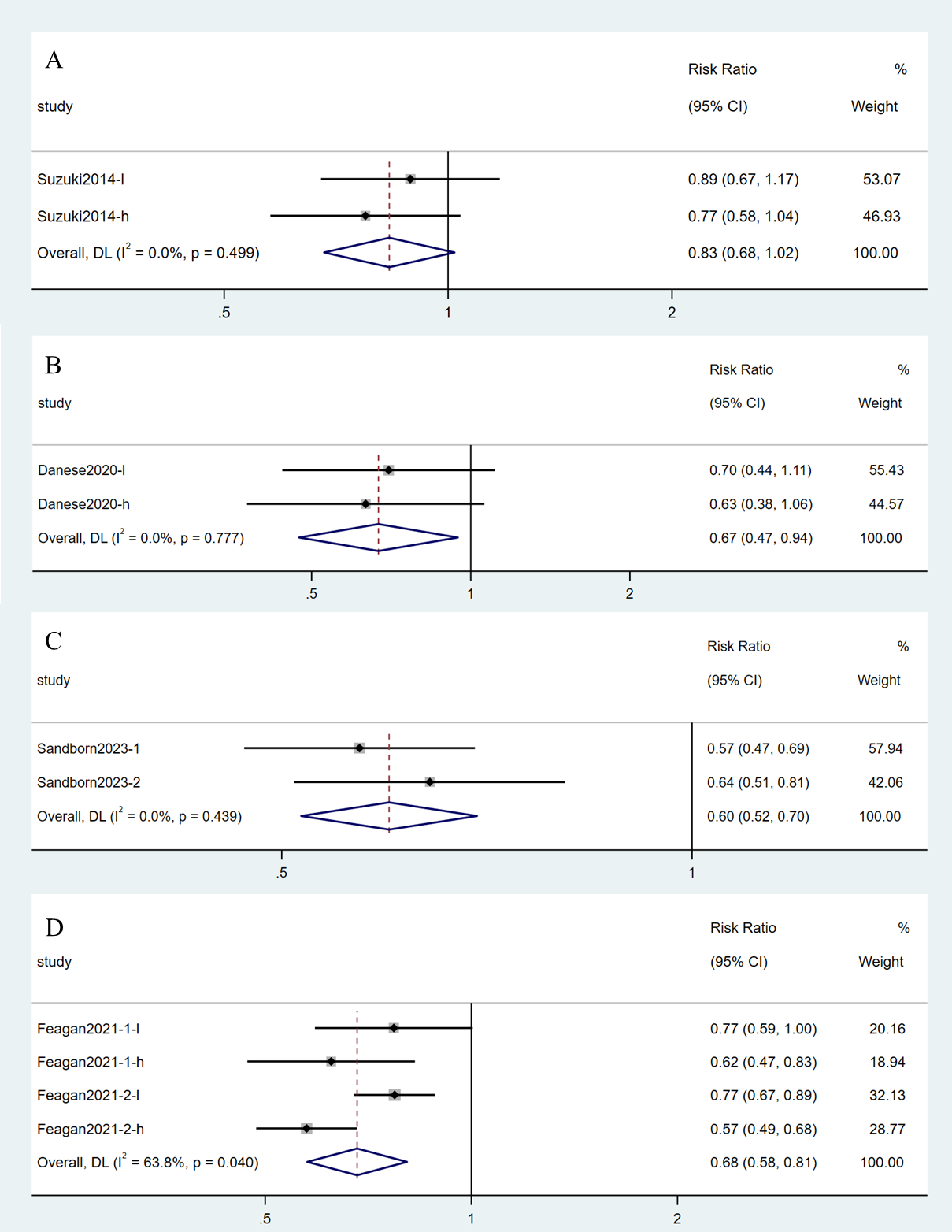

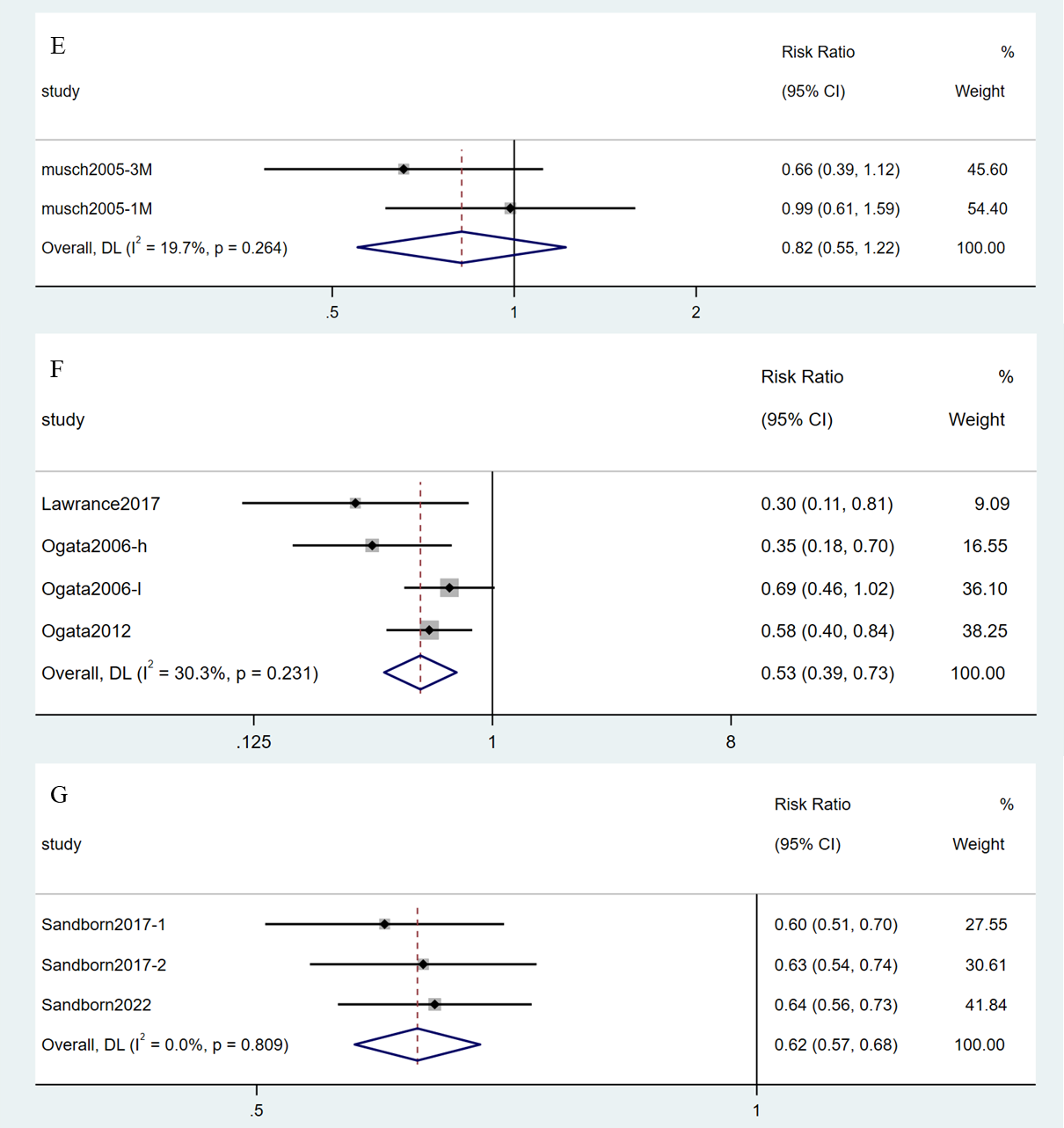
**

**Supplementary Figure 6 Forest plots and heterogeneity of head-to-head comparisons for clinical response. (A) adalimumab vs placebo; (B) apremilast vs placebo; (C) etrasimod vs placebo; (D) filgotinib vs placebo; (E) rIFN-β-1a vs placebo; (F) tacrolimus vs placebo; (G) tofacitinib vs placebo.**

**
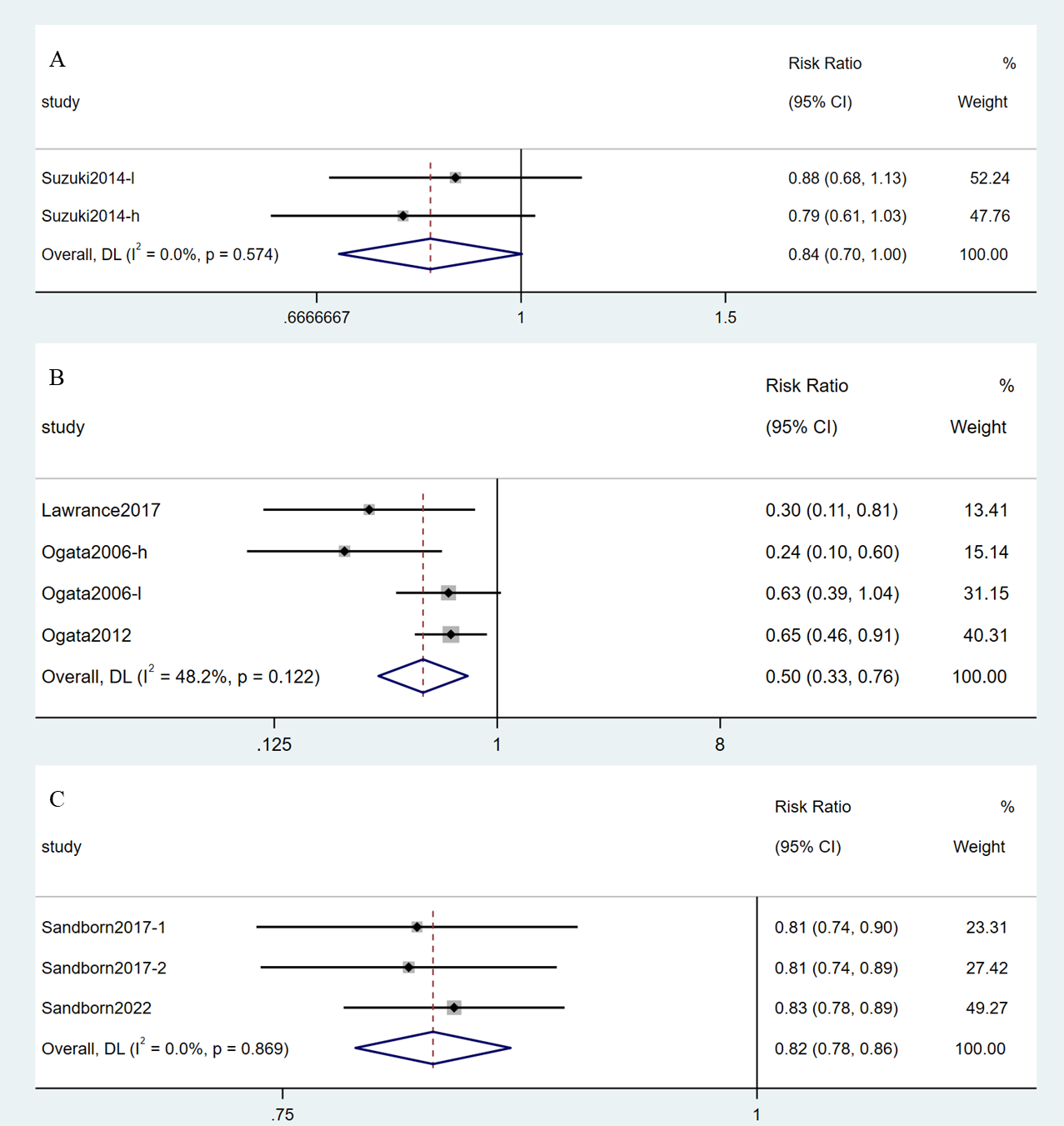
**

**Supplementary Figure 7 Forest plots and heterogeneity of head-to-head comparisons for mucosal healing. (A) adalimumab vs placebo;(B) tacrolimus vs placebo; (C) tofacitinib vs placebo.**

**
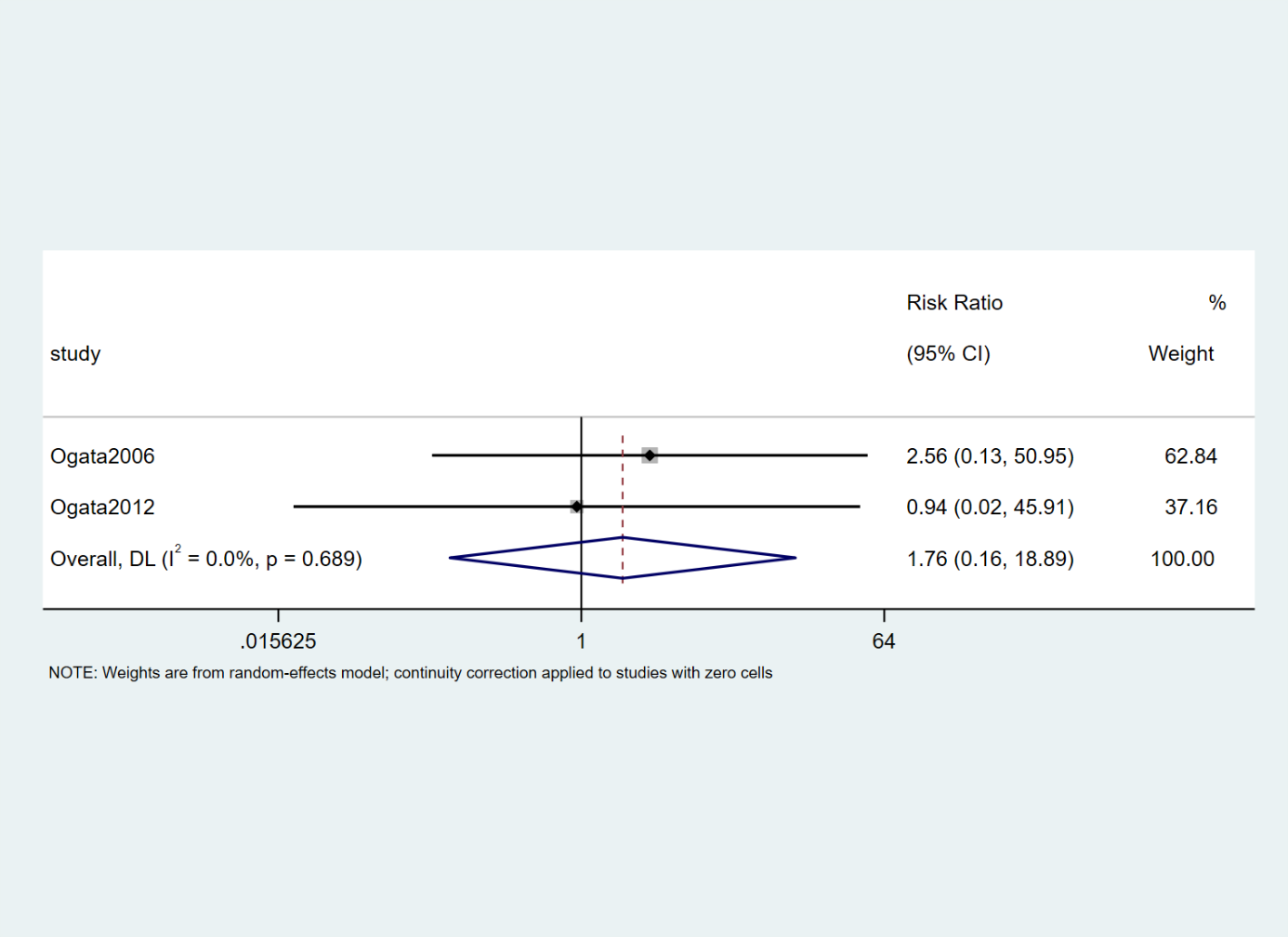
**

**Supplementary Figure 8** **Forest plots and heterogeneity of comparison of tacrolimus vs placebo for serious adverse events.**


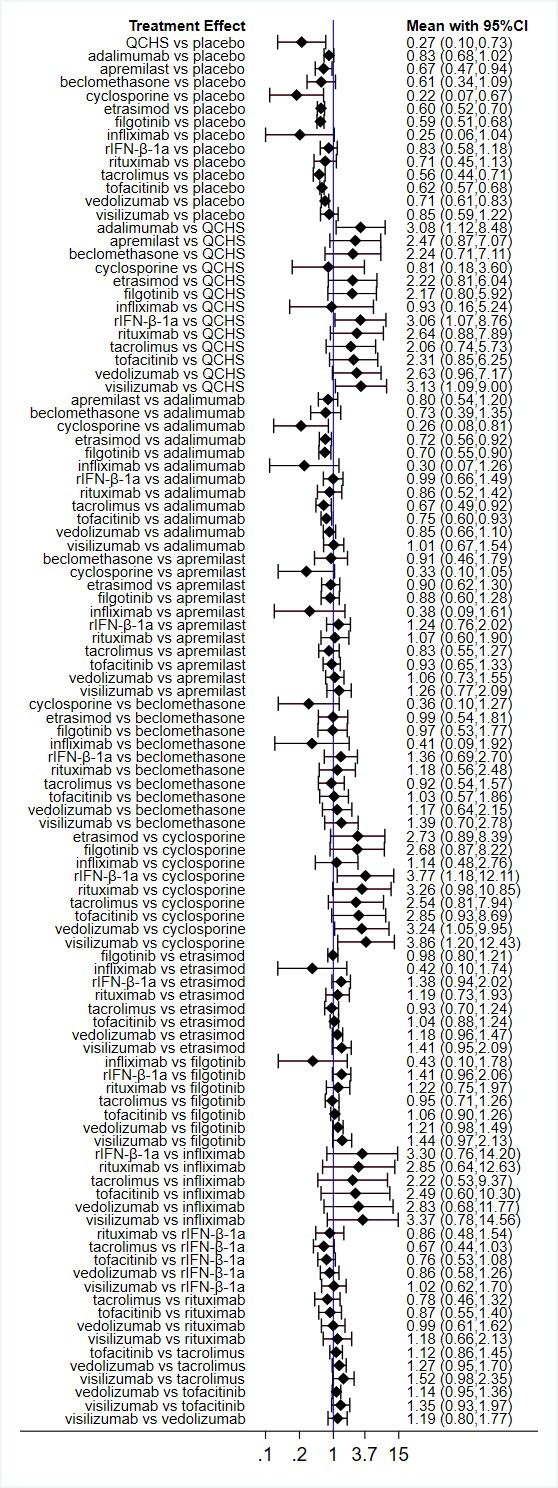

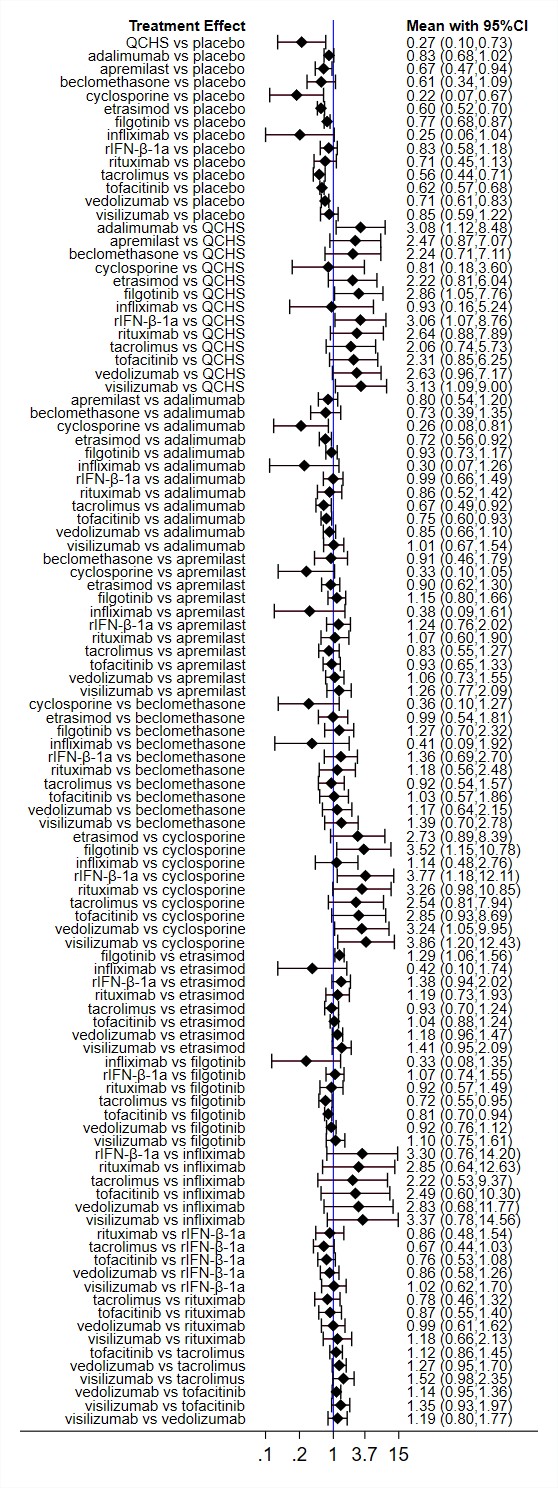


**A B**

**Supplementary Figure 9 Sensitivity analysis of filgotinib (A)high-dose group and (B) low-dose group.**

A

**
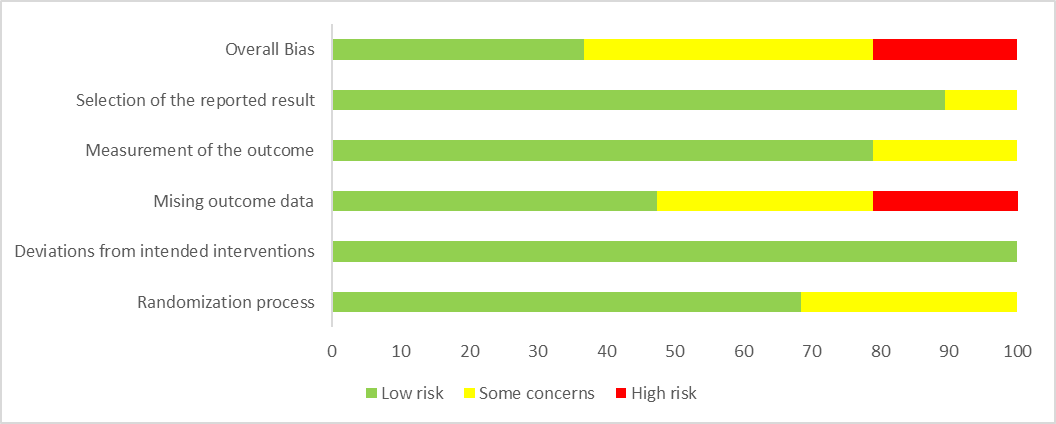
**

B

**Supplementary Figure 10 Results of risk of bias assessment using the Cochrane risk of bias tool 2. (A) risk of bias summary. (B) Risk of bias of the literature.**

**
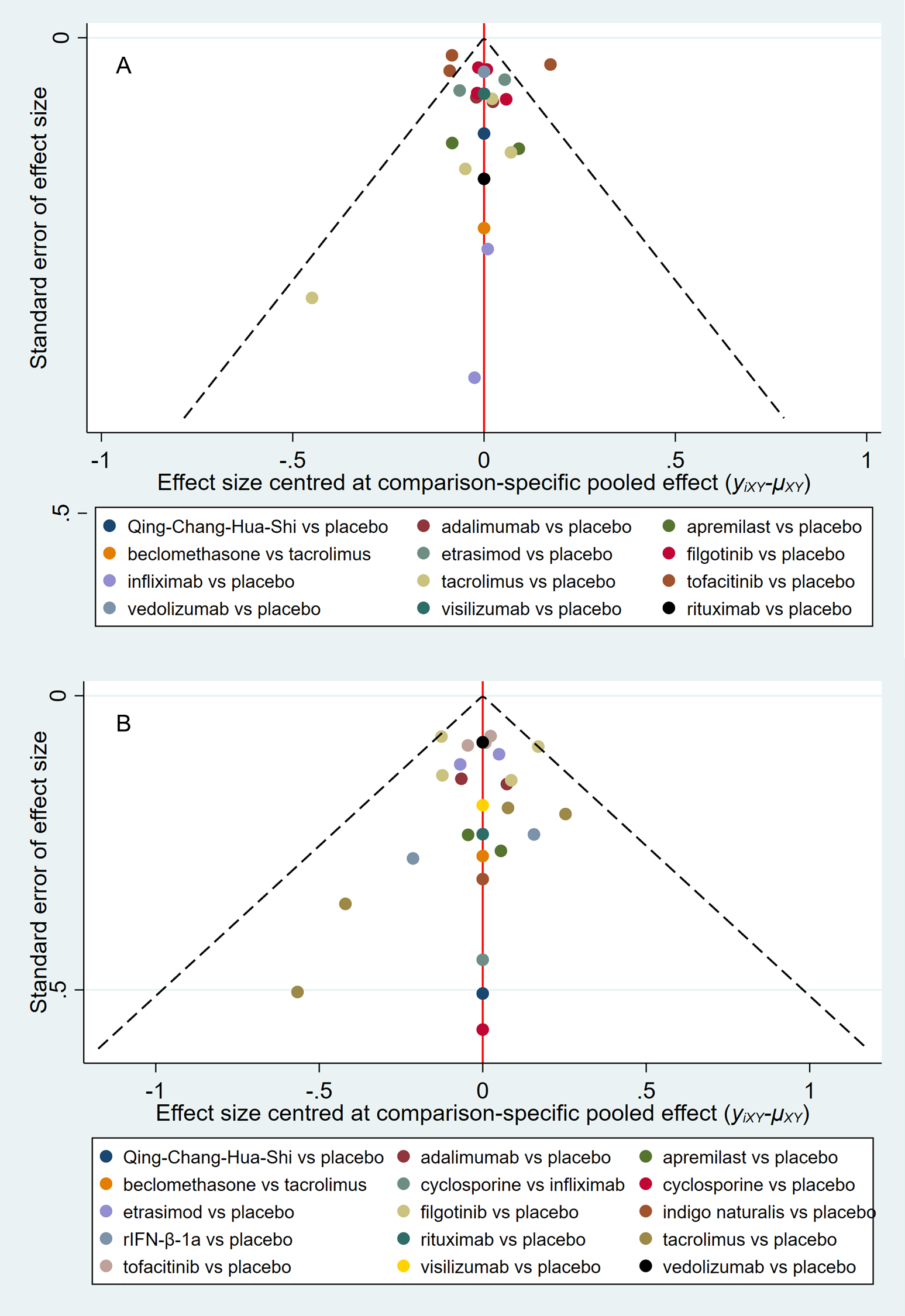
**

**
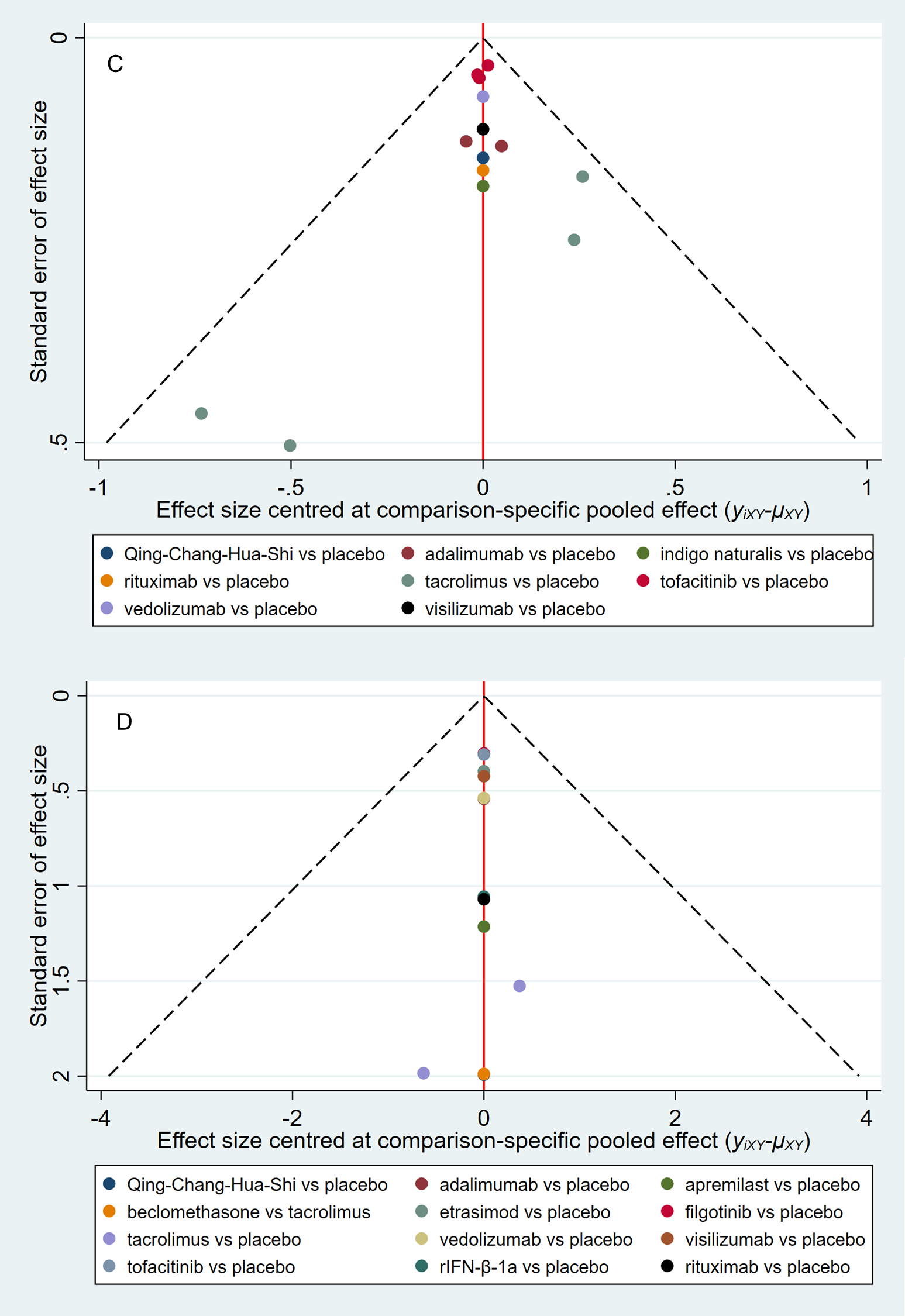
**

**Supplementary Figure 11** Publication bias of (A) remission studies, (B) response studies, (C) mucosal healing studies, and (D) serious adverse events studies.

**References:**

1. Danese S, Neurath MF, Kopoń A, et al. Effects of Apremilast, an Oral Inhibitor of Phosphodiesterase 4, in a Randomized Trial of Patients With Active Ulcerative Colitis. eng. *Clinical gastroenterology and hepatology : the official clinical practice journal*. 2020;18(11):2526-2534.e9. doi:10.1016/j.cgh.2019.12.032

2. Feagan BG, Rutgeerts P, Sands BE, et al. Vedolizumab as induction and maintenance therapy for ulcerative colitis. eng. *The New England journal of medicine*. 2013;369(8):699-710. doi:10.1056/NEJMoa1215734

3. Feagan BG, Danese S, Loftus EV, Jr., et al. Filgotinib as induction and maintenance therapy for ulcerative colitis (SELECTION): a phase 2b/3 double-blind, randomised, placebo-controlled trial. eng. *Lancet (London, England)*. 2021;397(10292):2372-2384. doi:10.1016/S0140-6736(21)00666-8

4. G Jr, Hertervig E, Friis-Liby I, et al. Infliximab as rescue therapy in severe to moderately severe ulcerative colitis: a randomized, placebo-controlled study. eng. *Gastroenterology*. 2005;128(7):1805-11. doi:10.1053/j.gastro.2005.03.003

5. Laharie D, Bourreille A, Branche J, et al. Ciclosporin versus infliximab in patients with severe ulcerative colitis refractory to intravenous steroids: a parallel, open-label randomised controlled trial. eng. *Lancet (London, England)*. 2012;380(9857):1909-15. doi:10.1016/S0140-6736(12)61084-8

6. Lawrance IC, Baird A, Lightower D, Radford-Smith G, Andrews JM, Connor S. Efficacy of Rectal Tacrolimus for Induction Therapy in Patients With Resistant Ulcerative Proctitis. Article. *Clinical Gastroenterology and Hepatology*. 2017;15(8):1248-1255. doi:10.1016/j.cgh.2017.02.027

7. Leiper K, Martin K, Ellis A, et al. Randomised placebo-controlled trial of rituximab (anti-CD20) in active ulcerative colitis. eng. *Gut*. 2011;60(11):1520-6. doi:10.1136/gut.2010.225482

8. Lichtiger S, Present DH, Kornbluth A, et al. Cyclosporine in severe ulcerative colitis refractory to steroid therapy. eng. *The New England journal of medicine*. 1994;330(26):1841-5. doi:10.1056/NEJM199406303302601

9. Lie M, Kreijne JE, Dijkstra G, et al. No Superiority of Tacrolimus Suppositories vs Beclomethasone Suppositories in a Randomized Trial of Patients With Refractory Ulcerative Proctitis. eng. *Clinical gastroenterology and hepatology : the official clinical practice journal*. 2020;18(8):1777-1784.e2. doi:10.1016/j.cgh.2019.09.049

10. Musch E, Andus T, Kruis W, et al. Interferon-beta-1a for the treatment of steroid-refractory ulcerative colitis: a randomized, double-blind, placebo-controlled trial. eng. *Clinical gastroenterology and hepatology : the official clinical practice journal*. 2005;3(6):581-6. doi:10.1016/s1542-3565(05)00208-9

11. Ogata H, Matsui T, Nakamura M, et al. A randomised dose finding study of oral tacrolimus (FK506) therapy in refractory ulcerative colitis. Article. *Gut*. 2006;55(9):1255-1262. doi:10.1136/gut.2005.081794

12. Ogata H, Kato J, Hirai F, et al. Double-blind, placebo-controlled trial of oral tacrolimus (FK506) in the management of hospitalized patients with steroid-refractory ulcerative colitis. Article. *Inflammatory bowel diseases*. 2012;18(5):803-808. doi:10.1002/ibd.21853

13. Probert CS, Hearing SD, Schreiber S, et al. Infliximab in moderately severe glucocorticoid resistant ulcerative colitis: a randomised controlled trial. eng. *Gut*. 2003;52(7):998-1002. doi:10.1136/gut.52.7.998

14. Sandborn WJ, Colombel JF, Frankel M, et al. Anti-CD3 antibody visilizumab is not effective in patients with intravenous corticosteroid-refractory ulcerative colitis. eng. *Gut*. 2010;59(11):1485-92. doi:10.1136/gut.2009.205443

15. Sandborn WJ, Su C, Sands BE, et al. Tofacitinib as Induction and Maintenance Therapy for Ulcerative Colitis. *The New England journal of medicine*. May 4 2017;376(18):1723-1736. doi:10.1056/NEJMoa1606910

16. Sandborn WJ, Peyrin-Biroulet L, Sharara AI, et al. Efficacy and Safety of Tofacitinib in Ulcerative Colitis Based on Prior Tumor Necrosis Factor Inhibitor Failure Status. Journal article. *Clinical gastroenterology and hepatology*. 2022;20(3):591‐601.e8. doi:10.1016/j.cgh.2021.02.043

17. Sandborn WJ, Vermeire S, Peyrin-Biroulet L, et al. Etrasimod as induction and maintenance therapy for ulcerative colitis (ELEVATE): two randomised, double-blind, placebo-controlled, phase 3 studies. eng. *Lancet (London, England)*. 2023;401(10383):1159-1171. doi:10.1016/S0140-6736(23)00061-2

18. Shen H, Zhang S, Zhao W, et al. Randomised clinical trial: Efficacy and safety of Qing-Chang-Hua-Shi granules in a multicenter, randomized, and double-blind clinical trial of patients with moderately active ulcerative colitis. eng. *Biomedicine & pharmacotherapy = Biomedecine & pharmacotherapie*. 2021;139:111580. doi:10.1016/j.biopha.2021.111580

19. Suzuki Y, Motoya S, Hanai H, et al. Efficacy and safety of adalimumab in Japanese patients with moderately to severely active ulcerative colitis. eng. *Journal of gastroenterology*. 2014;49(2):283-94. doi:10.1007/s00535-013-0922-y
